# Foods as Modulators of GLP-1 Secretion

**DOI:** 10.3390/nu18182995

**Published:** 2026-09-13

**Authors:** Halina Tkaczenko, Renata Kołodziejska, Anna Rymuszka, Piotr Kamiński, Natalia Kurhaluk

**Affiliations:** 1 Institute of Biology, Pomeranian University in Słupsk, K. Arciszewski St. 22B, 76-200 Slupsk, Poland; 2 Department of Medical Biology and Biochemistry, Collegium Medicum in Bydgoszcz, Nicolaus Copernicus University in Toruń, M. Karłowicz St. 24, 85-092 Bydgoszcz, Poland; renatak@cm.umk.pl; 3 Department of Physiology and Toxicology, Faculty of Medicine, The John Paul II Catholic University of Lublin, Konstantynów St. 1I, 20-708 Lublin, Poland; anna.rymuszka@kul.pl; 4 Department of Medical Biology and Biochemistry, Division of Ecology and Environmental Protection, Faculty of Medicine, Collegium Medicum in Bydgoszcz, Nicolaus Copernicus University in Toruń, M. Curie Skłodowska St. 9, 85-094 Bydgoszcz, Poland; piotr.kaminski@cm.umk.pl; 5 Department of Nature Conservation, Institute of Biological Sciences, Faculty of Biological Sciences, University of Zielona Góra, Prof. Z. Szafran St. 1, 65-516 Zielona Gora, Poland

**Keywords:** GLP-1, incretin biology, nutrient sensing, gut microbiota, fermented foods, postbiotics, polyphenols, GPR119, FFAR4, precision nutrition

## Abstract

**Background/Objectives**: Glucagon-like peptide-1 (GLP-1), which is released by enteroendocrine L-cells, is the primary incretin hormone and plays a pivotal role in glucose regulation, appetite control, and metabolic health. While GLP-1 receptor agonists offer significant therapeutic benefits, there is a growing interest in identifying foods that can increase endogenous GLP-1 production through physiological processes. However, the physiological impact of modulating GLP-1 through diet is modest compared with activating GLP-1 receptors pharmacologically. This narrative review summarizes the latest reports on how specific food matrices and gut microbiota-dependent pathways influence GLP-1 release, emphasizing their importance in precision nutrition. **Methods**: Literature published between 2001 and 2026 was reviewed, including experimental, clinical, and translational studies examining the effects of foods on GLP-1 secretion, L-cell activation, nutrient-sensing receptors, microbial metabolites, and metabolic outcomes. Evidence was synthesized for food categories such as fermented dairy products, fermented vegetables, whole grains, legumes, fiber-rich root vegetables, berries, grapes, green tea, turmeric and oily fish. **Results**: Foods modulate endogenous GLP-1 secretion through complementary pathways involving L-cell stimulation and the activation of receptors, including FFAR2, FFAR3, GPR119, TGR5 and FFAR4 (GPR120), as well as the production of microbial metabolites. Fermented dairy products, kimchi, kefir, Jerusalem artichokes, chicory root, oats, garlic, onions, berries, grapes, green tea and oily fish support incretin regulation through microbial fermentation, the generation of short-chain fatty acids (SCFAs), bile acid signalling and the release of bioactive compounds. These food matrices shape metabolite profiles more effectively than isolated nutrients, highlighting the importance of the interaction between whole foods and the gut microbiota. **Conclusions**: Foods rich in fermentable substrates, polyphenols, bioactive peptides and omega-3 fatty acids are promising dietary modulators of physiological GLP-1 activity. Taking into account microbiome characteristics, metabolic phenotype and individual postprandial responses could facilitate the development of personalized nutrition strategies to help prevent and manage obesity, type 2 diabetes and related cardiometabolic disorders.

## 1. Introduction

The earliest evidence defining the physiological role of glucagon-like peptide-1 (GLP-1) emerged in the late 1990s. In 1997, Nauck et al. [1] demonstrated that GLP-1 exerts a dominant inhibitory effect on gastric emptying, establishing gastrointestinal motility as a central determinant of postprandial glycaemia. Shortly afterwards, Hellström and Näslund (2001) [2] identified GLP-1 as a key component of the ileal brake, showing its ability to slow gastrointestinal transit, enhance satiety and reduce food intake. These early studies positioned GLP-1 as a hormone integrating appetite regulation, glucose control and gastrointestinal motility. A major conceptual advance was provided by Holst (2007) [3], who clarified that GLP-1 is produced by tissue-specific processing of proglucagon in intestinal L-cells and brainstem neurons, released in a biphasic pattern after meals and rapidly degraded by DPP-IV. GLP-1 acts both as an incretin, stimulating insulin secretion and suppressing glucagon secretion, and as an enterogastrone, slowing gastric emptying and stabilising postprandial glucose levels. These discoveries established the physiological basis for GLP-1’s role in glucose homeostasis, gastrointestinal motility and gut–brain communication. Building on these mechanistic insights, research in the early 2010s shifted towards analyzing how GLP-1 regulates appetite and feeding behavior.

Asmar et al. (2010) [4] demonstrated that GLP-1 reduces appetite, slows gastric emptying, and decreases food intake in humans. These effects occur primarily through the direct action of GLP-1 rather than via co-secreted peptides, such as amylin. Dailey and Moran (2013) [5] further demonstrated that, when released from L-cells, GLP-1 engages submucosal and myenteric neurons, vagal afferents and central nuclei in order to suppress food intake during and between meals. Shah and Vella (2014) [6] complemented this by reporting that GLP-1 delays gastric emptying while simultaneously acting on central appetite pathways. This indicates that its satiety effects arise from synergistic gut–brain signalling. Clinical observations following bariatric surgery provided additional support. Svane et al. (2016) [7] demonstrated that the markedly elevated postprandial levels of GLP-1 and PYY following Roux-en-Y gastric bypass surgery contribute to reduced appetite and weight loss. This emphasizes the physiological importance of endogenous GLP-1 in maintaining long-term energy balance.

The incretin system consists of two hormones: GLP-1, which is secreted by L-cells, and glucose-dependent insulinotropic polypeptide (GIP), which is released from K-cells. While both hormones enhance glucose-dependent insulin secretion, GLP-1 has broader metabolic effects, including reducing appetite, delaying gastric emptying and suppressing glucagon. In contrast, responses to GIP are often diminished in obesity and type 2 diabetes, and GIP may increase glucagon under certain metabolic conditions. These physiological differences are why GLP-1 is the primary incretin targeted in nutritional and pharmacological interventions, while K-cell signalling is less relevant for dietary modulation [1,2,3,4,5,6,7,8,9,10].

Alongside GLP-1, GIP is one of the two classical incretin hormones. Early human studies demonstrated its insulinotropic properties, showing that GIP stimulates insulin release following nutrient ingestion [11,12]. GIP secretion is strongly driven by dietary carbohydrates and fats, and its physiological relevance was recognized soon after the incretin concept was formulated [13]. However, the insulinotropic action of GIP is frequently diminished in obesity and type 2 diabetes, and under several metabolic conditions, GIP can promote glucagon secretion [14]. In contrast, GLP-1 maintains activity across metabolic states and exerts broader effects on appetite regulation, gastrointestinal motility, and glucose control. For these reasons, GLP-1 is the main nutritionally responsive incretin axis examined in this review.

The identification of GLP-1 as a regulator of energy homeostasis has led to the development of GLP-1 receptor agonists (GLP-1RAs). While GLP-1RAs can induce substantial weight loss, they are often discontinued due to gastrointestinal adverse effects, cost, the burden of injections and weight regain [15]. Observations from clinical practice indicate poor adherence, reduced dosages and diminished effectiveness outside of controlled trials, as demonstrated by Thomsen et al. (2025) [16]. These limitations highlight the need for strategies that activate gut–brain pathways through physiological nutrient sensing rather than pharmacological receptor stimulation.

Diet naturally activates enteroendocrine L-cells, the gut microbiota and the intestinal immune–metabolic interface [17,18,19]. Human nutrition studies indicate that foods rich in fermentable substrates, bioactive peptides, polyphenols and unsaturated lipids influence GLP-1 secretion through nutrient-sensing G-protein-coupled receptors expressed on L-cells [20,21,22]. Bodnaruc et al. (2016) [23] summarized how dietary composition affects pathways linked to incretin release, and further work shows that GLP-1 and GLP-2 support gut-barrier integrity and mediate communication between the microbiota and immune system [18]. Despite progress in understanding incretin physiology, current therapeutic frameworks rarely incorporate nutrition, microbial activity or nutrient-sensing pathways when modelling endogenous GLP-1 regulation. Ard et al. (2021) [8] noted that dietary patterns may interact with GLP-1 receptor agonists (GLP-1RAs), and comparative analyses suggest that variability in weight-loss outcomes may reflect differences in diet and microbial composition. Although these observations highlight the relevance of diet–microbiota interactions, the upstream routes through which food-derived compounds and microbial metabolites influence GLP-1 secretion remain insufficiently defined. Addressing this gap is the aim of the present review.

This review examines how selected food-derived systems—fermented foods, postbiotics, whey peptides, signalling amino acids and microbiota-generated lipid metabolites—activate the incretin axis through nutrient-sensing routes that differ from the direct receptor engagement produced by pharmacological GLP-1RAs. Agents such as semaglutide, liraglutide, dulaglutide and tirzepatide act through targeted GLP-1 receptor activation, generating predictable metabolic effects. In contrast, dietary systems influence enteroendocrine physiology at an earlier stage through microbial fermentation, postbiotic metabolites, bioactive lipids and peptide-driven epithelial signalling. These nutrient-dependent pathways demonstrate how GLP-1 secretion is shaped by nutritional and microbial factors that extend beyond receptor-targeted pharmacology.

This review advances current understanding by presenting a unified model of endogenous incretin regulation that integrates nutritional, microbial and metabolic perspectives. It explains how food-derived metabolites and microbial transformations affect GLP-1 physiology and identifies potential complementary targets for pharmacological therapies. Although dietary modulation produces weaker effects than receptor agonists, it exerts distinct regulatory actions on the intestinal environment that cannot be replicated by pharmacological agents. This observation aligns with earlier reports that nutrient-sensing processes in L-cells remain incompletely characterized, underscoring the importance of clarifying how diet influences incretin secretion.

## 2. Methods and Search Strategy

A structured literature search was conducted in PubMed, Scopus, Web of Science and Google Scholar to identify studies examining the modulation of endogenous GLP-1 secretion by diet, the sensing of nutrients by the gut microbiota, and precision nutrition approaches. The search covered the period from 2001 to 2026. The literature search was last updated in July 2026. Predefined keyword combinations and Boolean operators were used to conduct searches in PubMed, Scopus, Web of Science and Google Scholar. This identified 308 records, which were then screened for relevance based on their titles, abstracts and full texts. After removing duplicates and applying the inclusion and exclusion criteria, 308 studies were found to be eligible and included in the qualitative synthesis.

To ensure methodological transparency, in line with SANRA Item 3, the search strategy was expanded to include targeted queries for each dietary category and receptor pathway discussed in Section 4. The search terms therefore comprised both broad and domain-specific keywords. Broad terms included: “GLP-1”, “endogenous GLP-1 secretion”, “dietary modulation”, “enteroendocrine L-cells”, “gut microbiota”, “short-chain fatty acids”, “fermentable fibre”, “precision nutrition” and “incretin physiology”, which are all important topics in this field. Targeted searches were also performed for mechanistic pathways and nutrient classes that were central to the review’s narrative structure.

These included: bile acids and bile-acid signalling (“bile acids”, “FXR”, “TGR5”, “bile acid–microbiota interactions”) to support Section 4. In order to support Section 4, the consumption of fermented foods and postbiotic metabolites is recommended. These include “fermented foods”, “postbiotics”, “microbial metabolites” and “exopolysaccharides”. Protein-derived peptides and amino-acid signalling (“whey peptides”, “protein hydrolysates”, “amino acid sensing”, “GPR119”) were included to support Section 4. Polyphenols and plant bioactives (such as “polyphenols”, “microbial biotransformation”, “plant bioactives” and “intestinal epithelial signalling”) were included to support Section 4. Omega-3 fatty acids and lipid-sensing pathways (“omega-3 fatty acids”, “EPA”, “DHA”, “GPR120”, “FFAR4”, “β-arrestin signalling”) were included to support Section 5.7.

The reference lists of key articles and relevant reviews were screened to identify additional sources. As this review synthesizes evidence from the fields of mechanistic, microbial and nutritional science, it was conducted as a narrative review rather than a systematic review or meta-analysis.

Studies examining the influence of specific nutrients, whole foods, dietary patterns or microbial metabolites on endogenous GLP-1 secretion through direct nutrient sensing or microbiota-dependent mechanisms were included, as were studies providing mechanistic, preclinical or clinical insights into L-cell activation, microbial fermentation or host–microbe metabolic signalling. Studies focusing exclusively on pharmacological GLP-1 receptor agonists without a nutritional or microbial context were excluded, as were articles lacking mechanistic relevance and non-peer-reviewed materials. When multiple publications addressed the same biological mechanism, priority was given to recent studies with high-quality experimental evidence, while seminal publications were retained for historical context.

After study selection, the reports were organized according to the biological pathways through which dietary components modulate endogenous GLP-1 secretion. These pathways included SCFA-mediated FFAR2/FFAR3 activation, peptide-dependent GPR119 signalling, lipid-dependent GPR120 activation, bile-acid-dependent TGR5 signalling and microbial biotransformation of polyphenols. This structure facilitated comparison across diverse nutritional interventions and highlighted shared signalling routes relevant to enteroendocrine physiology. The preparation and reporting of this review followed SANRA recommendations to ensure clarity, transparency, and coherence.

## 3. Historical Foundations and Primary Discovery of Incretin Hormones

The idea that the intestine contributes to glucose regulation stems from early observations made in 1906, when researchers in Liverpool reported that intestinal extracts reduced blood glucose levels. Interest in this phenomenon resurfaced in the 1960s when several research groups demonstrated that oral glucose produced a stronger insulin response than intravenous glucose at matched glycemia. This established the physiological basis for the incretin effect (as reviewed by Tschöp and Friedman, 2023 [24]). Creutzfeldt later formalized the criteria for defining incretin hormones, describing them as gut-derived factors released in response to nutrients that are capable of stimulating insulin secretion only under hyperglycemic conditions [13]. Contemporary analyses summarize these historical developments and highlight their clinical implications, including the emergence of GLP-1-based therapeutics and the recognition of GLP-1 biology through major scientific awards (as reviewed in [24,25,26,27]).

The first peptide to fulfill these criteria was glucose-dependent insulinotropic polypeptide (GIP). Foundational work in the early 1970s provided evidence of its existence, and it was isolated from porcine intestine in 1973. Subsequent experimental studies have shown that GIP is a potent stimulator of postprandial insulin secretion; however, its therapeutic potential in the treatment of diabetes has been limited [11,12]. These findings established GIP as the first incretin hormone and framed the search for additional gut-derived peptides with similar properties. Advances in molecular endocrinology have clarified the structure of the proglucagon precursor, revealing that it encodes multiple biologically active peptides. In 1982, Lund and his team demonstrated that pancreatic preproglucagon contains two glucagon-related coding sequences arranged in tandem [28]. Building on this work, Bell and colleagues cloned the hamster preproglucagon gene in 1983, revealing conserved peptide sequences across species and providing the molecular basis for identifying glucagon-related peptides [29].

Post-translational processing of proglucagon was shown to differ between the pancreas and the intestine. In 1986, Mojsov and colleagues demonstrated that intestinal processing generates GLP-1(7–37), thus establishing the molecular identity of the peptide that would later be recognized as an incretin hormone [30]. Earlier radioimmunoassay studies had already demonstrated the nutrient-stimulated release of glucagon-related peptides from the intestine [31], thus supporting the idea that a glucagon gene product could act as an incretin. In 1984, Mojsov and Merrifield developed improved methods for synthesizing mammalian glucagon, enabling the generation of peptide variants used in early biological assays and supporting the functional characterization of glucagon-derived peptides [32]. The physiological relevance of GLP-1 was experimentally established in 1987 when Mojsov, Weir and Habener demonstrated that GLP-1(7–37) potently stimulates insulin secretion in the perfused rat pancreas [33]. This work provided the first direct evidence that GLP-1 acts as an incretin hormone, establishing its role as a central regulator of postprandial insulin secretion.

For decades, GIP was regarded as an ineffective insulinotropic agent with limited therapeutic potential, a view shaped by early physiological studies showing modest insulinotropic activity and minimal clinical relevance in diabetes [11,12]. This perception persisted even as methodological advances improved the measurement of incretin hormones and clarified their postprandial dynamics [34]. However, the landscape changed dramatically with the emergence of multi-agonist therapies. The clinical success of tirzepatide and the development of next-generation triple agonists targeting GLP-1, GIP and glucagon receptors has renewed interest in GIP physiology and prompted a re-evaluation of its metabolic actions [14]. These therapeutic breakthroughs reflect a broader evolution in incretin science, synthesized in contemporary analyses that trace the trajectory from foundational discovery to modern pharmacology (as reviewed in [24,25,26,27]).

## 4. Nutrient Sensing and Microbiota-Dependent Regulation of GLP-1

Over the past decade, our scientific understanding of GLP-1 as a metabolic regulator has grown considerably [35,36,37,38,39,40,41,42,43,44,45,46,47,48]. This has revealed its pivotal function in glucose homeostasis, appetite control and gastrointestinal physiology [3,5,6,37,38,39,40,41,42]. Initial nutritional studies showed that GLP-1 is synthesised by enteroendocrine L-cells and released in response to nutrient intake [21,22,43]. Its secretion is influenced by direct interactions between dietary components and G-protein-coupled receptors on the apical surface of these cells [47,48], and reviewed in [23]. Research into intestinal physiology has also revealed that GLP-1 plays a role in maintaining barrier integrity, modulating inflammation, and coordinating communication between the gut, immune system, and microbiota [44,45,46,47,48,49,50,51].

**Figure 1:**
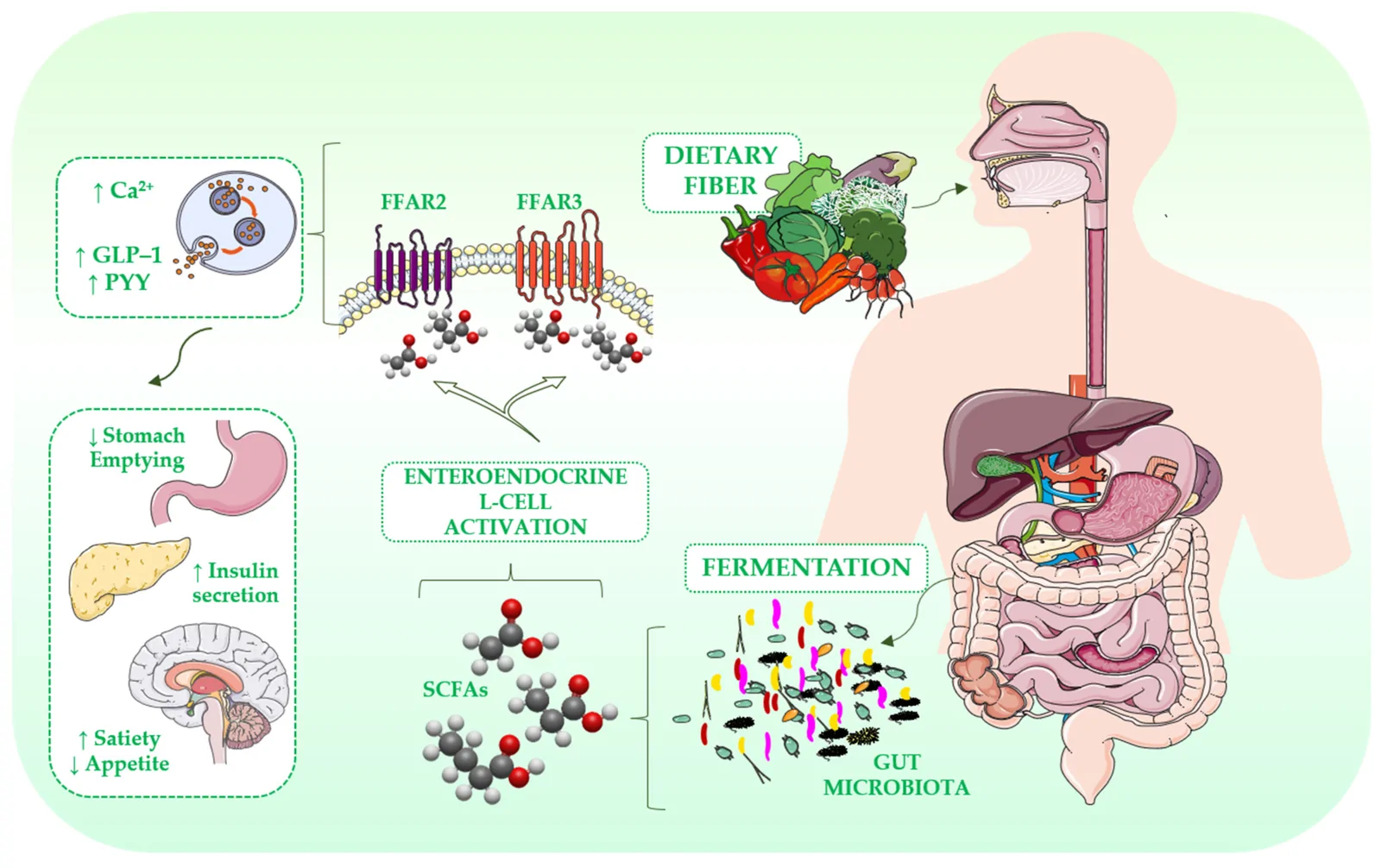
Metabolic dialogue between diet, intestinal microbiota, enteroendocrine cells and hypothalamic appetite circuits. Fermentation of fiber by intestinal microbiota leads to the formation of short-chain fatty acids (SCFAs: acetate, propionate and butyrate), which activate FFAR2 (GPR43) and FFAR3 (GPR41) receptors on enteroendocrine cells. This increases intracellular Ca^2+^, inducing exocytosis of secretory granules and enhancing GLP-1 and PYY secretion. GLP-1 increases insulin secretion, improves glucose tolerance, slows gastric emptying, increases satiety and reduces appetite. It also acts centrally, stimulating POMC/CART neurons and inhibiting NPY/AgRP neurons in the hypothalamus to reinforce satiety and reduce food intake. GIP and glucagon engage in overlapping hypothalamic pathways that are involved in energy balance and contribute to the integrated regulation of appetite and metabolism. Image provided by Servier Medical Art (https://smart.servier.com/), licensed under CC BY 4.0 (https://creativecommons.org/licenses/by/4.0/, accessed on 10 July 2026). Abbreviations: FFAR2—free fatty acid receptor 2; FFAR3—free fatty acid receptor 3; GLP-1—glucagon-like peptide-1; PYY—peptide YY; SCFAs—short-chain fatty acids. Upward arrows (↑) indicate increased levels, secretion, or activity, whereas downward arrows (↓) indicate decreased activity or physiological responses.

These findings establish GLP-1 as a pivotal integrator of nutrient sensing, intestinal function, and metabolic regulation, as well as an incretin hormone. These functions position enteroendocrine L-cells at the interface between luminal nutrient availability and whole-body metabolic homeostasis, enabling rapid physiological adaptation to changes in dietary composition (Figure 1).

Observations suggest that shifts in microbial metabolism induced by diet influence GLP-1 secretion [23,41,46,49]. Short-chain fatty acids produced during fermentation interact with L-cell receptors and contribute to nutrient-dependent incretin release [47,48,50]. Foods that supply fermentable substrates, unsaturated lipids and amino acids support these processes by shaping microbial activity and epithelial signalling, as summarized previously in references [38,42,43,44]. These observations demonstrate that microbial fermentation and nutrient sensing are connected processes that influence endogenous GLP-1 secretion, establishing the gut environment as a pivotal signalling interface [41,51].

These discoveries have shifted the focus of incretin biology from individual signalling pathways to an integrated gut–microbiota–host communication network. As Bodnaruc et al. (2016) [23] reviewed, dietary composition influences the secretion of endogenous GLP-1 through nutrient-sensing receptors expressed on L-cells. This influences appetite, glucose homeostasis, and gastrointestinal motility via the gut–brain–pancreas axis. Meanwhile, clinical reviews have documented the metabolic efficacy of GLP-1 receptor agonists [35,36,37,38,39]. Wang et al. (2023) [9] demonstrated that these agents mimic the actions of endogenous GLP-1 to reduce food intake, slow gastric emptying, and improve glycemic control. Meanwhile, Moiz et al. (2025) [10] emphasized the expanding therapeutic scope of these agents across cardiometabolic and neurological conditions. These contemporary findings reinforce the view that GLP-1 is a central integrator of appetite regulation, glucose homeostasis and gastrointestinal motility. At the same time, they make clear that nutritional modulation of endogenous GLP-1 secretion and pharmacological receptor activation are complementary approaches to improving metabolic health, rather than competing ones.

Recent advances in metabolic science have further strengthened the rationale for targeting endogenous GLP-1 secretion through personalized dietary strategies, based on this integrated concept [41,46,50,51,52]. Research into the biology of enteroendocrine cells suggests that the function of L cells in obesity and type 2 diabetes is dynamic rather than declining uniformly. Early metabolic dysfunction is characterized by a compensatory phase involving increased L-cell density and enhanced GLP-1 secretion. This reflects an adaptive response to emerging insulin resistance. As the condition progresses towards overt diabetes, however, this compensation diminishes, transitioning into reduced L-cell density, impaired nutrient sensing, and attenuated GLP-1 responsiveness [53,54,55]. Personalized nutritional strategies are most effective when endogenous hormone responsiveness is still preserved [19]. Reviews indicate that high-fiber foods, nuts, avocados and eggs can support GLP-1 secretion and metabolic improvements [23]. Variability in microbiota composition, habitual diet and metabolic phenotype substantially affects the magnitude of GLP-1 responses to identical nutritional stimuli. These observations support a precision-nutrition framework in which dietary composition, microbial ecology and nutrient-sensing pathways are aligned to enhance endogenous GLP-1 secretion and promote long-term metabolic health [56].

Understanding the biology of enteroendocrine L-cells is fundamental to nutritional science, as these highly specialized cells play a key role in communicating dietary components, microbial metabolites and host metabolic pathways [21,44]. L-cells are the main physiological source of GLP-1 and are therefore the central focus of this review. Their distribution along the distal gut, their extensive nutrient-sensing repertoire, and their direct responsiveness to ingested food position them as the key interface between dietary stimuli and GLP-1 secretion [57]. The importance of the enteroendocrine pathway is supported by foundational work demonstrating that intestinal L-cells process preproglucagon via the proconvertase 1/3 pathway to generate GLP-1 [28,29,30,32]. Subsequent studies have established GLP-1 as a potent stimulator of insulin release [11,12,33], forming the basis of the modern incretin concept [13] and ultimately enabling the development of GLP-1-based therapies [24,25,26,58].

In contrast, the presence of GLP-1 in α-cells reflects a context-dependent response associated with glucagon signalling and metabolic stress, rather than being a significant contributor to circulating levels. Although α-cells are abundant within the islet and play an important role in glucose homeostasis, their primary product remains glucagon, generated through proconvertase 2 processing of preproglucagon [59]. Under specific metabolic conditions, including diabetes, α-cells can generate small amounts of GLP-1 and exert local paracrine effects on insulin secretion [60]. However, this response remains limited and does not replace the dominant enteroendocrine route.

Therefore, the physiological actions of GLP-1 in regulating glucose homeostasis are predominantly driven by L-cell secretion [61], forming the nutritionally relevant axis examined in this manuscript. As chemosensory endocrine cells, L-cells continuously monitor the intestinal lumen, translating nutritional and microbial cues into coordinated hormonal responses that regulate gastrointestinal function and systemic metabolic homeostasis. Developing precision nutrition strategies that enhance endogenous GLP-1 secretion requires detailed knowledge of L-cell location and the detection and integration of luminal signals from nutrients, the gut microbiota, the enteric nervous system and circulating hormones [18,46,62]. In this section, review articles published between 2001 and 2026 are used solely to summarize data showing how L-cell physiology has been characterized across primary studies, while acknowledging that the foundational anatomical and functional discoveries were made in the 1970s and early 2000s [28,29,30,32].

Radioimmunoassay studies by Bryant and Bloom [17] revealed distinct regional patterns in the distribution of gut hormones. They showed that vasoactive intestinal polypeptide (VIP) is present at high concentrations throughout the bowel, whereas secretin, gastric inhibitory polypeptide (GIP), motilin and cholecystokinin (CCK) are predominantly localized in the proximal small intestine. Pancreatic glucagon was almost entirely confined to the pancreas, while enteroglucagon exhibited a broad distribution, with maximal levels observed in the ileum. These findings provided the first quantitative evidence for hormone-specific localization within the gastrointestinal tract [17]. Building on this physiological framework, which is characterized by the nutrient-sensing properties of L-cells, includes the expression of SGLT1 and KATP channels, which enable the rapid detection of luminal carbohydrates and the initiation of hormone secretion. Genetic studies showing that loss of transcription factors such as Foxa1, Foxa2 and Neurogenin-3 disrupts enteroendocrine differentiation provide further support for the central role of L-cells in metabolic regulation. This provides a strong biological rationale for targeting them through nutritional interventions [63].

The functional versatility of L-cells is supported by their broad repertoire of nutrient-sensing receptors [64,65]. As reviewed in [18], L-cells detect carbohydrates, proteins and lipids through membrane receptors and transport systems, enabling rapid secretion of GLP-1 and PYY after food ingestion [66,67,68,69]. An overview in reference [22] describes how amino acids stimulate hormone release by activating receptors such as the calcium-sensing receptor (CaSR) and GPRC6A [70]. This leads to membrane depolarization, intracellular calcium signalling, and exocytosis of secretory granules [71]. However, experimental evidence shows that robust GLP-1 secretion induced by basic L-amino acids does not require GPRC6A. For example, oral administration of L-arginine or L-ornithine elicits strong GLP-1 release in both wild-type and GPRC6A-knockout mice [72], demonstrating that the dominant pathway operates independently of this receptor. Lipid-derived signals are detected through receptors including GPR120, whereas bile-acid-dependent signalling converges on the farnesoid X receptor (FXR), which directly represses GLP-1 secretion and remodels L-cell metabolic programming [73]. Additionally, intestine-restricted FXR activation using fexaramine shapes the gut microbiota and enhances TGR5-dependent GLP-1 secretion, thereby improving glucose tolerance and promoting adipose tissue browning [74]. These pathways operate as an integrated receptor network that enables L-cells to interpret various nutritional cues and generate endocrine responses that reflect the composition of ingested food.

In line with the dietary focus of this review, the section highlights foods known to influence GLP-1 secretion. Fermented dairy products, fermented vegetables, fiber-rich foods such as chicory and Jerusalem artichokes, and whole grains including oats, berries, grapes, garlic, onions, green tea, and marine fish provide bioactive compounds, fermentable substrates, and lipid mediators that engage L-cell receptors [18,22,75]. These foods affect GLP-1 release through short-chain fatty acid (SCFA) formation, bile-acid-dependent signalling, peptide generation and lipid-mediated receptor activation, providing physiologically relevant dietary routes for incretin modulation. Recent studies show that L-cells operate within broader neuroendocrine and microbial networks. Ren et al. (2024) [52] demonstrated that sympathetic fibers form close anatomical contacts with L-cells and suppress GLP-1 secretion via α2-adrenergic signalling. Venkataraman et al. (2025) [54] identified an estrogen-responsive paracrine circuit linking PYY-expressing L-cells with neighboring serotonergic enterochromaffin cells. This circuit amplifies intestinal sensitivity through SCFA-dependent upregulation of Olfr78. These findings illustrate how dietary factors, microbial metabolites, neural inputs and hormonal signals converge to regulate endogenous GLP-1 secretion.

At the intracellular level, L-cells contain signalling structures that coordinate the detection of nutrients with the release of hormones. Humbert et al. (2026) [55] showed that endoplasmic reticulum–mitochondria contact sites act as hubs integrating nutrient sensing, calcium homeostasis and GLP-1 exocytosis. Atanga et al. (2023) [76] proposed that L-cell receptors, ion channels, and transcriptional regulators offer multiple points for modulating endogenous incretin release. Cellular characterization by Kuhre et al. (2021) [53] provides the structural context for understanding how dietary signals are translated into endocrine responses. These intracellular features may contribute to inter-individual variability in GLP-1 secretion following dietary interventions. Thus, the evidence shows that L-cells function within interconnected nutritional, microbial and neuroendocrine networks that support metabolic homeostasis. Their ability to link nutrient sensing with microbial metabolism, autonomic regulation, and intracellular signalling establishes them as pivotal mediators in the connection between dietary composition and endogenous GLP-1 secretion.

The fermentation of dietary fiber produces short-chain fatty acids (SCFAs), which act as key signalling metabolites that regulate endogenous GLP-1 secretion [21,44]. Tolhurst et al. [77] showed that acetate, propionate and butyrate directly stimulate GLP-1 release from colonic L-cells by activating FFAR2 and FFAR3, thereby increasing intracellular Ca^2+^. Deletion of either receptor impairs SCFA-induced GLP-1 secretion and glucose tolerance. These findings established the FFAR2/FFAR3 axis as a central link between microbial fermentation and incretin signalling. Further evidence from perfused colon models and controlled propionate infusion demonstrates that SCFAs also stimulate PYY secretion and modulate orexigenic gene expression, thereby influencing satiety pathways [47,49]. Metabolites derived from the microbiota account for many of the metabolic benefits associated with dietary patterns, as highlighted by Kouraki et al. (2026) [46]. FFAR2 senses acetate and propionate preferentially via Gq and Gi/o signalling pathways, whereas FFAR3 responds more strongly to propionate and butyrate through Gi/o pathways [20,48,78]. Hunt et al. (2024) confirmed SCFA-driven FFAR2/FFAR3 activation in perfused colon preparations, thereby emphasizing their metabolic importance [79]. FFAR3 also detects ketone bodies, suggesting that L-cells integrate both microbial and host-derived metabolic cues [80].

Dietary interventions can also modulate this axis by altering microbial composition and receptor expression. For example, Dendrobium officinale polysaccharides increase SCFA-producing taxa, elevate intestinal SCFA levels, upregulate FFAR2/FFAR3, and enhance GLP-1 secretion in prediabetic mice [45]. Similarly, *Lactobacillus acidophilus* restores microbial balance and increases colonic GLP-1 and FFAR2/FFAR3 expression [80]. The fermentation of dietary fiber produces short-chain fatty acids (SCFAs), which act as key signalling metabolites that regulate endogenous GLP-1 secretion [21,44]. Deletion of either receptor impairs SCFA-induced GLP-1 secretion and glucose tolerance. These findings established the FFAR2/FFAR3 axis as a central link between microbial fermentation and incretin signalling. The diet–microbiota–SCFA–FFAR2/FFAR3–GLP-1 pathway is one of the best characterized mechanisms through which dietary fiber influences appetite regulation, glucose homeostasis, and energy balance, supporting precision nutrition strategies aimed at enhancing endogenous GLP-1 secretion [15,81,82].

Traditionally associated with lipid digestion, bile acids also function as endocrine signalling molecules that regulate glucose homeostasis, appetite, and intestinal physiology [83,84,85]. Their composition and activity are influenced by diet and microbial biotransformation, forming a bile acid–microbiota–L-cell axis that connects dietary habits to enteroendocrine output. Thomas et al. (2009) demonstrated that activation of the bile acid receptor TGR5 stimulates intestinal GLP-1 secretion and improves glucose tolerance, thereby redefining bile acids as active metabolic signals [86]. Dietary patterns that modify bile acid pools, including high-fiber diets and foods rich in plant bioactives [87,88], can therefore influence GLP-1 release through TGR5-dependent pathways.

**Figure 2:**
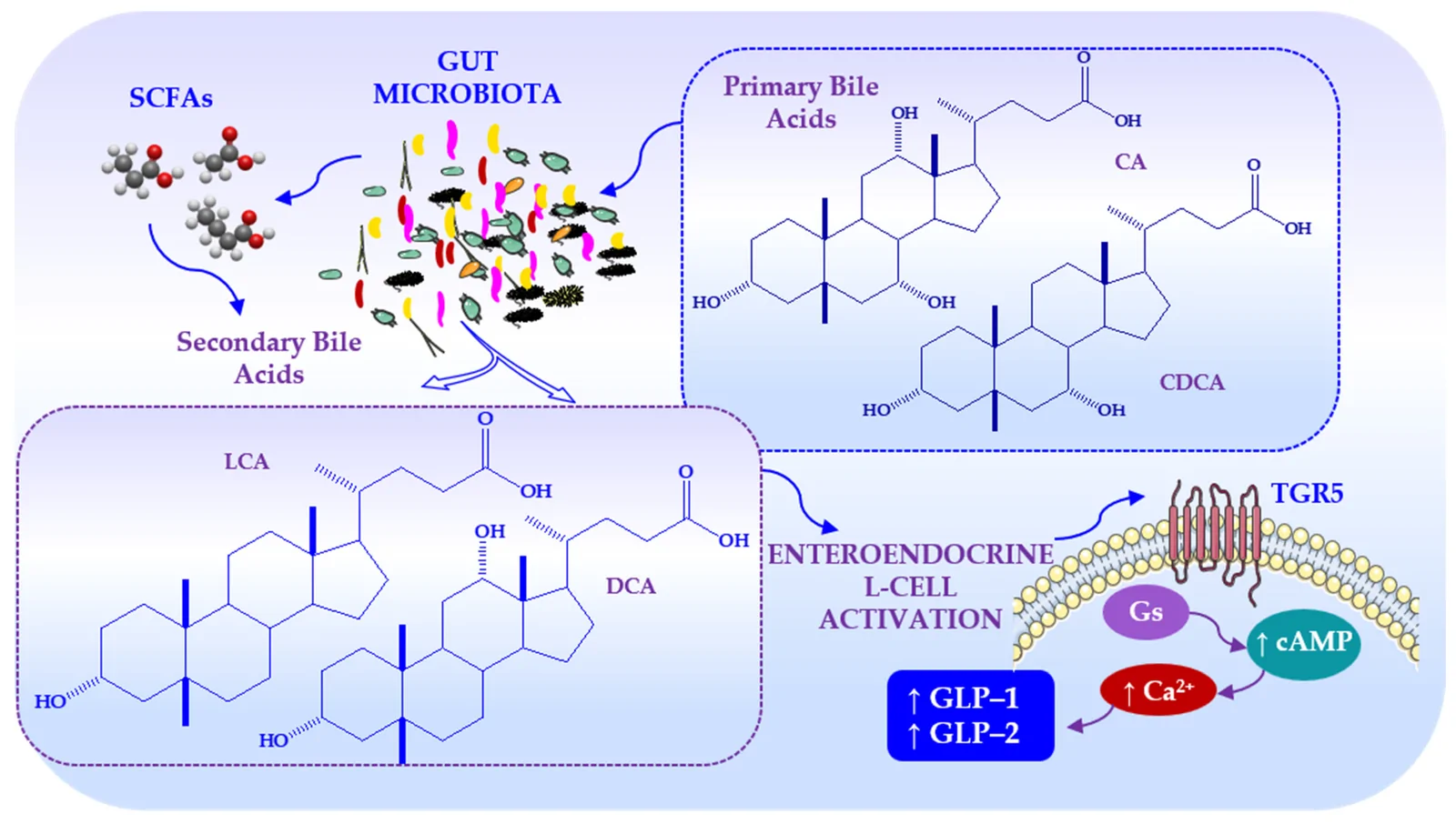
The bile acid–microbiota–TGR5–GLP-1 axis as a mechanism of incretin secretion regulation. Primary bile acids synthesized in the liver are enzymatically converted by the intestinal microbiota into secondary bile acids, primarily deoxycholic acid (DCA) and lithocholic acid (LCA). Secondary bile acids activate the TGR5 receptor (GPBAR1) located on enteroendocrine L cells, which initiates an intracellular signaling pathway dependent on Gs protein, adenylate cyclase, cAMP, and increased Ca^2+^ concentration, leading to exocytosis of secretory granules and increased secretion of the incretin hormones GLP-1 and GLP-2. GLP-1. Image provided by Servier Medical Art (https://smart.servier.com/), licensed under CC BY 4.0 (https://creativecommons.org/licenses/by/4.0/, accessed on 10 July 2026). Abbreviations: CA—cholic acid; cAMP—cyclic adenosine monophosphate; CDCA—chenodeoxycholic acid; DCA—deoxycholic acid; GLP-1—glucagon-like peptide-1; GLP-2—glucagon-like peptide-2; Gs—stimulatory G protein; LCA—lithocholic acid; SCFAs—short-chain fatty acids; TGR5—Takeda G protein-coupled receptor 5, GPBAR1. Upward arrows (↑) indicate increased levels.

TGR5 signalling operates within a multi-organ metabolic network involving the gut, liver, and endocrine pancreas. Potthoff et al. (2013) showed that colesevelam reduces hepatic glucose production by enhancing GLP-1 action via TGR5-mediated mechanisms, indicating that bile acid manipulation can modulate systemic glucose metabolism [89]. Hunt et al. (2020) confirmed TGR5 expression in L-cells and demonstrated increased GLP-1 and GLP-2 secretion upon receptor activation, thereby linking bile acid signalling to mucosal adaptation and metabolic regulation [90]. Nutritional interventions also shape this pathway. Li et al. (2023) reported that red ginseng extracts activate intestinal TGR5, elevate GLP-1 levels, and improve high-fat-diet-induced insulin resistance [91]. Together, these findings demonstrate that dietary components and bioactive compounds can modulate bile acid signalling via microbiota-dependent transformations and receptor-mediated mechanisms (Figure 2).

The clinical relevance of bile acid–TGR5 signalling is highlighted by its role in metabolic surgery, multi-organ endocrine regulation, and host–microbiota interactions [91]. McGavigan et al. (2017) showed that vertical sleeve gastrectomy increases circulating bile acids, enhances nutrient-stimulated GLP-1 secretion, and improves glycemic control via TGR5-dependent mechanisms [92]. TGR5 activation in pancreatic α-cells beyond the intestine modulates glucagon release and augments GLP-1-mediated insulin secretion, as demonstrated by Kumar et al. (2016) [93]. Herbal and microbiota-targeted therapies also influence this axis: For example, Lingguizhugan Decoction increases non-12-OH bile acids, which are potent TGR5 agonists, thereby elevating GLP-1 and thermogenesis [94]. Conversely, dysbiosis alters bile acid composition and disrupts gut–immune–metabolic signalling in post-transplant diabetes [95]. Dual-target molecules combining TGR5 agonism with SSTR5 antagonism can enhance GLP-1 secretion while limiting gallbladder-related adverse effects [96]. Clinical evidence from Roux-en-Y gastric bypass further confirms that bile acid remodelling improves metabolic outcomes via TGR5-dependent GLP-1 pathways [97]. Thus, these findings suggest that bile acid–microbiota interactions are a key regulatory axis that integrates dietary signals, microbial metabolism, and endocrine responses across various tissues.

Enteroendocrine cells sense dietary lipids and peptides through a network of GPCRs, including GPR119, GPR120 (FFAR4), GPR40 (FFAR1), GPR41 (FFAR3) and GPR43 (FFAR2). These receptors translate lipid-derived metabolites into hormonal signals that regulate appetite, insulin secretion, and inflammatory pathways [20,98,99]. Therefore, they act as a nutrient-sensing interface that links dietary lipid composition to enteroendocrine function and whole-body metabolic homeostasis [22,78,100,101].

The first insights into lipid-sensing pathways emerged from studies demonstrating that enteroendocrine and islet cells express G-protein-coupled receptors (GPCRs) capable of detecting long-chain fatty acids and lipid-derived metabolites [102,103,104]. Early research identified GPR40 as a receptor activated by medium- and long-chain fatty acids, which elevates intracellular Ca^2+^ and potentiates glucose-stimulated insulin secretion in beta cells [105,106]. Subsequent studies have confirmed its role in regulating nutrient-dependent insulin release [103,105,107]. In parallel, GPR120 was identified as an omega-3 fatty acid receptor with anti-inflammatory and insulin-sensitizing effects [100,108] shifting lipid biology towards receptor-mediated metabolic signalling.

As research progressed, it became clear that lipid sensing is a shared property of enteroendocrine cells throughout the gut. Cho and Kieffer (2010) showed that nutrient-stimulated GIP secretion involves SGLT1, GPR40, GPR120 and GPR119, revealing functional overlap between K- and L-cells [109]. Nguyen et al. (2012) [110] demonstrated that long-chain fatty acids activate GPR40 and GPR120, whereas lipid-derived ethanolamides signal through GPR119. Reimann and Gribble (2016) [21] confirmed that both L- and K-cells express GPR40 and GPR120, secreting GLP-1 and GIP in response to long-chain fatty acids. Ekberg et al. (2016) [111] identified GPR119 as a sensor of triglyceride-derived metabolites that acts synergistically with GPR40 to enhance incretin secretion. Thus, these findings suggest that GPR119 and GPR120 are central components of the enteroendocrine nutrient-sensing system that drives diet-induced GLP-1 release.

**Figure 3:**
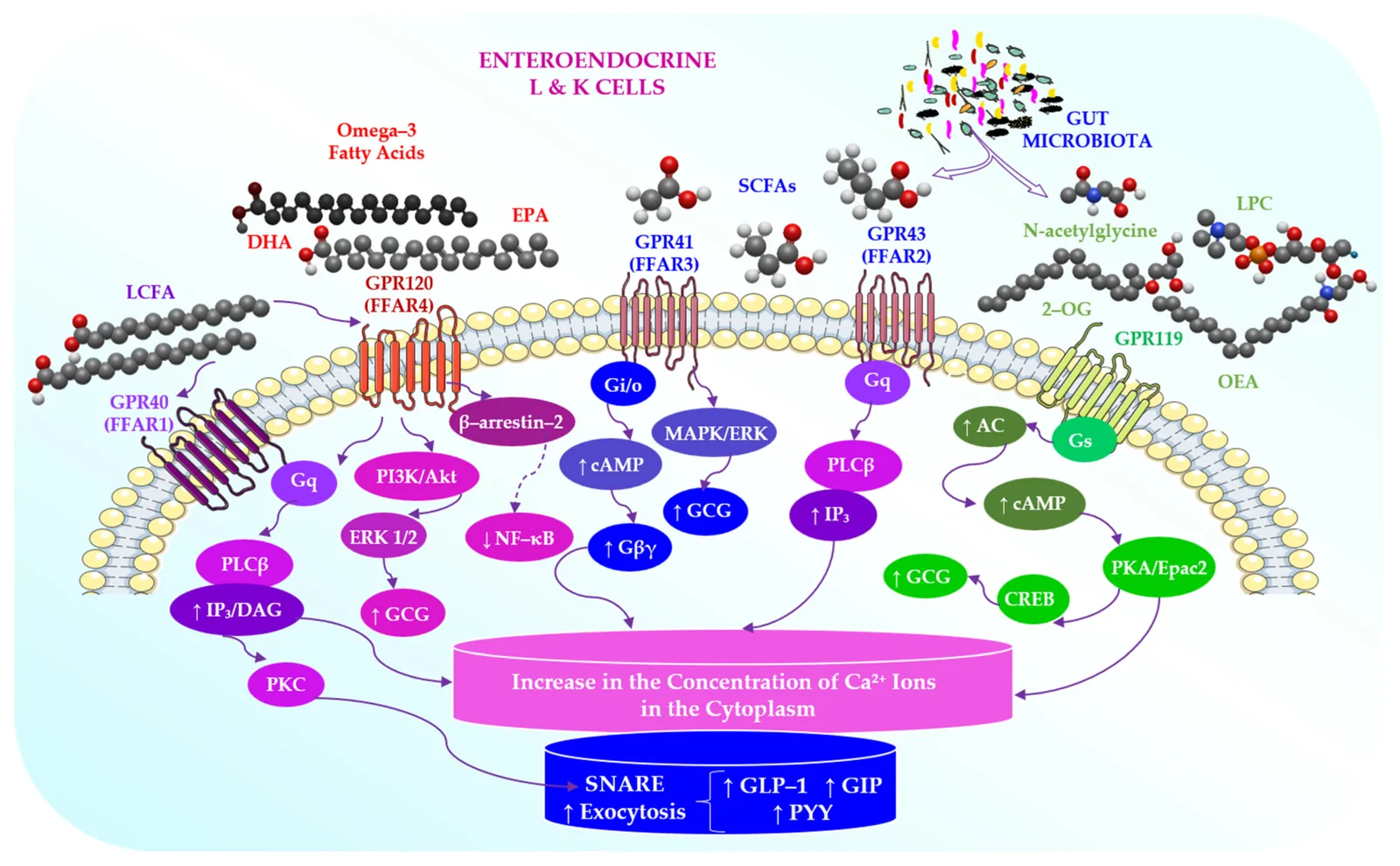
GPCRs in L and K enteroendocrine cells as mediators of the effects of lipids and microbiota metabolites on GLP-1 secretion. Long-chain fatty acids activate the GPR40 (FFAR1) and GPR120 (FFAR4) receptors, while triglyceride metabolites and ethanolamides are ligands for the GPR119 receptor. Short-chain fatty acids (SCFAs), produced by fiber fermentation by gut microbiota, activate the GPR41 (FFAR3) and GPR43 (FFAR2) receptors. The microbiota produces bioactive lipid metabolites, such as *N*-acylglycine and lysophosphatidylcholine (LPC), which modulate the activity of selected GPCRs. Receptor activation leads to the stimulation of G protein (Gq, Gs, or Gi)-dependent signaling pathways, including activation of phospholipase Cβ (PLCβ), adenylate cyclase, increased cAMP concentration, activation of protein kinase A (PKA), Epac protein, protein kinase C (PKC), and the PI3K/Akt and MAPK/ERK pathways. The combined effect of these processes is an increase in cytoplasmic Ca^2+^ concentration, activation of the SNARE exocytic complex, and enhanced secretion of GLP-1, GIP, and PYY. Long-term activation of selected receptors also increases GCG gene expression, enhancing GLP-1 biosynthesis. Image provided by Servier Medical Art (https://smart.servier.com/), licensed under CC BY 4.0 (https://creativecommons.org/licenses/by/4.0/, accessed on 10 July 2026). Abbreviations: 2-OG—2-oleoylglycerol; AC—adenylyl cyclase; Akt—protein kinase B, PKB; cAMP—cyclic adenosine 3′,5′-monophosphate; CREB—cAMP response element-binding protein; DAG—diacylglycerol; DHA—docosahexaenoic acid; EPA—eicosapentaenoic acid; Epac-2—exchange protein directly activated by cAMP 2; ERK1/2—extracellular signal-regulated kinases 1/2; Gβγ–G beta-gamma subunits; GCG—glucagon gene; Gi/o—inhibitory G protein; GIP—glucose-dependent insulinotropic polypeptide; GLP-1—glucagon-like peptide-1; GPR40 (FFAR1)—free fatty acid receptor 1; GPR41 (FFAR3)—free fatty acid receptor 3; GPR43 (FFAR2)—free fatty acid receptor 2; GPR119—G protein-coupled receptor 119; GPR120 (FFAR4)—free fatty acid receptor 4; Gs—stimulatory G protein; Gq—Gq protein; IP_3_—inositol 1,4,5-trisphosphate; LCFA—long-chain fatty acids; LPC—lysophosphatidylcholine; MAPK—mitogen-activated protein kinase; NF-κB—nuclear factor kappa B; OEA—oleoylethanolamide; PI3K—phosphoinositide 3-kinase; PKA—protein kinase A; PKC—protein kinase C; PLCβ—phospholipase C beta; PYY—peptide YY; SCFAs—short-chain fatty acids. Solid arrows indicate the direction of signalling or activation; upward arrows (↑) indicate increased activity or secretion, downward arrows (↓) indicate decreased activity or inhibition; and dashed arrows indicate proposed or indirect signalling interactions.

The therapeutic relevance of lipid-sensing G-protein-coupled receptors (GPCRs) is increasingly recognized in metabolic disease, inflammation, and cardiometabolic risk. Evidence summarized in reference [112] shows that deleting GPR119 or GPR120 impairs glucose tolerance and exacerbates inflammation. Scheen (2016) [113] identified GPR40, GPR119 and GPR120 as promising targets for insulin secretagogues, while Moran et al. (2016) [114] highlighted their roles in hormone biosynthesis, secretion and cell survival. Kiepura et al. (2021) demonstrated the anti-inflammatory and anti-atherosclerotic effects of GPR120 activation [115]. Ghislain and Poitout (2021) emphasized that multiple free fatty acid receptors, including GPR40, GPR41, GPR43 and GPR120, act as integrated lipid sensors, whereas GPR119 primarily responds to lipid-derived metabolites [116]. Thus, these receptors form a coordinated nutrient-sensing network that links dietary lipid composition to enteroendocrine function and whole-body metabolic homeostasis (Figure 3).

Recent work shows that lipid-sensing GPCRs also detect microbiota-derived lipid metabolites, linking diet, microbial metabolism and enteroendocrine signalling. Onodera et al. (2017) [117] demonstrated that the EPA-derived metabolite 5-HEPE activates regulatory T cells via GPR119 and GPR120. Meanwhile, Raka et al. (2019) described GPR40 and GPR120 as sensors of medium- and long-chain fatty acids, and GPR119 as responsive to lipid derivatives shaped by diet and microbial metabolism [118]. In 2024, Drzazga et al. [51] made a significant breakthrough by revealing that bacterial *N*-acyl glycines can trigger the activation of GPR40, GPR55, GPR119 and GPR120, leading to an increased secretion of GLP-1. In a further study, Szustak et al. (2024) [119] demonstrated that insulin secretion is regulated through GPR119 by palmitoleic-acid-enriched lysophosphatidylcholines. The results suggest that these receptors, which are sensitive to lipids, work together as a network to detect both dietary and microbiota-derived lipids. This, in turn, helps to coordinate GLP-1 release, insulin sensitivity, and inflammatory tone. Their ability to recognize structurally diverse lipid species positions GPR119, GPR120 and related receptors as promising targets for precision-nutrition strategies aimed at improving metabolic health [120,121].

## 5. Dietary Components Enhancing Endogenous GLP-1 Secretion

### 5.1. Fermentable Fibers and Resistant Starch

Fermentable fibers such as inulin, fructans and resistant starch (RS)—which are naturally present in legumes, whole grains, potatoes, sweet potatoes, green bananas and cooked-and-cooled starchy foods—are well-established enhancers of endogenous GLP-1 secretion [122,123,124]. These substrates reach the colon intact, where they are fermented by microbes to generate SCFAs that activate FFAR2/FFAR3 signalling in L-cells. Consistent findings from rodent studies show that RS increases GLP-1 and PYY through SCFA-dependent mechanisms [123,125], and elevated GLP-1 contributes to improved insulin sensitivity [126]. Human trials confirm the translational relevance, showing that fermentable carbohydrates increase postprandial GLP-1 and improve glucose tolerance [127,128]. Therefore, these findings establish fermentable fibers as effective modulators of microbiota–enteroendocrine interactions.

Randomized clinical trials provide consistent evidence that inulin improves metabolic health, primarily by modulating gut microbiota activity and increasing the production of SCFAs. In healthy adults, acute inulin supplementation significantly increased circulating acetate, propionate and butyrate levels, while reducing postprandial free fatty acid and ghrelin concentrations. It also transiently increased GLP-1, suggesting a potential role in regulating appetite and preventing type 2 diabetes [129]. Similarly, long-term supplementation with wheat-derived fermentable fiber enhanced SCFA production and increased GLP-1 secretion after nine to twelve months in hyperinsulinemic individuals, indicating that sustained colonic adaptation may contribute to improved insulin sensitivity and reduced diabetes risk [130]. A randomized, double-blind, placebo-controlled trial in patients with type 2 diabetes showed that inulin, particularly when combined with sodium butyrate, significantly improved fasting blood glucose, waist circumference, diastolic blood pressure and GLP-1 concentrations. This highlights the therapeutic potential of inulin-based interventions in managing metabolic disorders [128].

These effects are driven by the fermentation of indigestible carbohydrates by colonic microbes, leading to the production of SCFAs—primarily acetate, propionate and butyrate—which function as signalling metabolites rather than simple energy substrates. Notably, SCFAs activate FFAR2 and FFAR3 receptors on enteroendocrine L-cells, stimulating the secretion of GLP-1 and PYY [78,102]. Evidence from portal metabolite flux studies [129,130] and clinical work on inulin [131] suggests that the strength of hormonal responses depends on the composition of SCFAs and the kinetics of their absorption in the intestine [132,133]. Further studies by Bodinham et al. (2013) [134] and Boll et al. (2016) [135] have demonstrated that the fermentability of different fiber fractions, including arabinoxylan oligosaccharides (AXOS), resistant starch and cereal fibers, varies, as does their ability to modulate GLP-1 secretion. In rodent models, fermentation-driven endocrine effects also extend to central appetite regulation, as demonstrated by increased hypothalamic proopiomelanocortin (POMC) expression [136]. These findings indicate that dietary fibers influence both peripheral hormone secretion and central pathways involved in energy homeostasis. To provide a structured overview of these heterogeneous findings, Table 1 summarizes the evidence from human, rodent and porcine studies.

Thus, fermentable fibers and resistant starch exert metabolic benefits through colonic fermentation, SCFA production and FFAR2/FFAR3 activation on L-cells, consistently enhancing GLP-1 and PYY secretion and improving appetite and glucose regulation across animal and human studies. These observations highlight the potential of fiber-driven SCFA signalling as a reliable dietary approach to stimulate endogenous incretins, establishing fermentable carbohydrates as pivotal regulators of GLP-1 biology in precision nutrition strategies.

### 5.2. Food Sources Rich in Fermentable Fibers and Resistant Starch

Foods that are naturally rich in fermentable fibers and resistant starch are one of the dietary approaches that have been most extensively investigated for enhancing endogenous GLP-1 secretion. Unlike pharmacological GLP-1 receptor agonists, these foods indirectly stimulate incretin secretion by modifying the intestinal luminal environment, promoting microbial fermentation, and activating nutrient-sensing pathways in enteroendocrine L-cells. Consequently, they offer a strategy that integrates diet, gut microbiota and host metabolism [122,124,143].

The best-characterized nutritional modulators of endogenous GLP-1 secretion are inulin-type fructans (ITFs). Early experimental studies demonstrated that chicory-derived ITFs undergo colonic fermentation, generating SCFAs that activate FFAR2 and FFAR3 receptors on enteroendocrine L-cells and stimulate GLP-1 release [122]. As ITFs selectively promote the growth of beneficial saccharolytic bacteria, they enhance both microbial ecology and enteroendocrine signalling, thereby strengthening the gut–microbiota–GLP-1 axis. Further human intervention studies have demonstrated that Jerusalem artichoke, a natural source of ITFs, reduces postprandial glycemia and significantly lowers GIP concentrations in a dose-dependent manner [143,144]. A recent meta-analysis of 32 randomized controlled trials supports these findings, showing that chicory-derived ITFs reduce body weight, fat mass and waist circumference, thus confirming their clinically relevant metabolic benefits [143]. Notably, the consumption of foods naturally rich in ITFs, such as chicory root, Jerusalem artichoke, onions and garlic, enables the achievement of these effects through habitual dietary intake rather than supplementation alone.

Resistant starch (RS) is a key component of the second major class of nutritional GLP-1 enhancers, operating via different mechanisms. As RS is not digested in the small intestine, it reaches the colon, where microbial fermentation produces acetate, propionate and butyrate. This stimulates the secretion of GLP-1 and PYY via the activation of FFAR2/FFAR3 [123,125,131]. Animal studies have shown that RS consumption leads to reductions in adiposity and improvements in insulin sensitivity [125,145]. Human crossover trials indicate that chilled potatoes, which contain substantially higher amounts of resistant starch than freshly cooked potatoes, reduce early postprandial glucose, insulin and GIP responses [146]. Similarly, incorporating non-homogenized cooked vegetables into a rice meal was shown to slow carbohydrate digestion and improve postprandial glucose response in both in vitro and in vivo tests. The texture properties of the vegetables may play an important role in the underlying mechanisms of glycemic control [147]. The resistant starch content of foods can be substantially increased by simple culinary practices such as cooking followed by cooling. This makes it a practical and inexpensive nutritional strategy with considerable translational potential [148]. Additional dietary sources, including oats and green banana flour, naturally contain resistant starch and may have similar fermentation-dependent endocrine effects [149,150,151].

Several plant-derived foods also influence endogenous GLP-1 secretion through pathways that do not rely on SCFA formation. Polyphenol-rich foods and culinary spices interact directly with enteroendocrine nutrient-sensing pathways, and also influence oxidative stress, inflammatory signalling, and microbial metabolism [152,153,154,155]. One study involving human participants demonstrated that consuming a polyphenol-rich curry increased postprandial GLP-1 concentrations in a dose-dependent manner [152]. Further experimental studies showed that bioactive compounds derived from onions and apples improve inflammatory and vascular biomarkers while modulating metabolic hormone secretion [154]. Similarly, sweet potato leaf extract, which is rich in caffeoylquinic acids, has been shown to enhance GLP-1 secretion and attenuate hyperglycemia in experimental models [156]. These findings suggest that polyphenols may complement fermentation-dependent mechanisms by directly influencing L-cell signalling and improving the intestinal metabolic environment.

Protein hydrolysates are a distinct class of nutritional GLP-1 enhancers that differ from fermentable fibers in that they stimulate incretin secretion via bioactive peptide signalling rather than microbial fermentation [157]. Both rice and wheat protein hydrolysates have been shown to stimulate GLP-1 secretion in enteroendocrine cell models and to improve glucose tolerance in vivo [158,159]. Some peptide fractions also inhibit dipeptidyl peptidase-4 (DPP-4), thereby increasing the proportion of biologically active GLP-1 available for receptor activation [160]. These dual mechanisms—enhanced hormone secretion and reduced hormone degradation—distinguish protein hydrolysates from fiber-based nutritional strategies, making them attractive candidates for developing functional foods that target postprandial metabolic control [154]. Thus, current evidence shows that inulin-type fructans, resistant starch, polyphenol-rich foods and specific protein hydrolysates boost the body’s own GLP-1 activity by working in different ways, including signalling SCFAs, activating GPCRs, modulating incretin secretion and inhibiting GLP-1 degradation [161,162,163,164]. These nutritional strategies offer a biologically sound, non-pharmacological approach to improving glucose regulation, appetite control and metabolic health.

Table 2 summarizes the biological, clinical and nutritional characteristics of the four product groups, integrating evidence from human, animal and in vitro studies.

These findings demonstrate that the secretion of endogenous GLP-1 can be enhanced through several complementary nutritional mechanisms. These include the microbial fermentation of fermentable fibres, the activation of FFAR2/FFAR3 by SCFAs, the direct stimulation of nutrient-sensing receptors by plant bioactives and the enhancement of GLP-1 secretion and stability by peptides. Rather than acting through a single pathway, these dietary components engage with multiple interconnected biological processes that, collectively, improve glucose homeostasis, appetite regulation, and metabolic health.

### 5.3. Fermented Foods and Postbiotics

Fermented foods represent a unique nutritional category because, as well as being sources of nutrients, they are also complex biological matrices containing microbial metabolites that can modulate enteroendocrine signalling and host metabolism. The International Scientific Association for Probiotics and Prebiotics (ISAPP) defines fermented foods as “foods made through desired microbial growth and enzymatic conversion of food components” [177]. This definition directly links microbial activity with the production of bioactive compounds that affect gastrointestinal physiology. During fermentation, microorganisms transform carbohydrates, proteins, and phytochemicals into bioactive compounds such as SCFAs, organic acids, bioactive peptides, exopolysaccharides, vitamins, and structurally modified polyphenols. This markedly increases the biochemical complexity and functional properties of foods [176,178,179]. Sharma et al. (2020) [180] emphasized that fermentation plays a vital role in enhancing the nutritional, sensory and functional properties of foods, highlighting the formation of metabolites that are increasingly recognized as modulators of nutrient sensing, gut hormone secretion and metabolic homeostasis. Consequently, fermented foods such as kefir, yoghurt, kimchi and other traditional fermented products should be viewed as dietary systems capable of influencing endogenous GLP-1 secretion through multiple complementary mechanisms, not merely as probiotic carriers [181,182].

The physiological effects of fermented foods extend beyond the delivery of viable microorganisms, as many of their biological activities are mediated by fermentation-derived metabolites that remain active regardless of microbial viability. This concept is reflected in the ISAPP consensus definition of postbiotics, which describes them as “preparations of inanimate microorganisms and/or their components that confer a health benefit to the host” [183]. The postbiotic concept provides a valuable framework for understanding the interaction between microbial cell fragments, extracellular vesicles, cell wall components, and fermentation-derived metabolites with intestinal epithelial cells and enteroendocrine L-cells [184,185]. Many postbiotic molecules activate nutrient-sensing pathways directly or indirectly, modifying microbial ecology, bile acid metabolism, and epithelial signalling to create a favorable environment for endogenous GLP-1 secretion [186,187]. Caffrey et al. (2025) [188] further emphasized the need for “clinically relevant tools for identifying and consuming fermented foods”, highlighting that the accurate characterization of the composition of fermented foods is essential for linking specific postbiotic signatures with physiological outcomes, including incretin secretion.

Current data suggest that the metabolic effects of fermented foods stem from the combined action of various fermentation-derived metabolites rather than individual compounds acting alone [189]. Computational and systems biology analyses performed by Jimoh and Adebo (2025) [190] demonstrated that these metabolites have considerable anti-obesogenic potential and interact with signalling pathways involved in glucose regulation, insulin sensitivity, and lipid metabolism. Many of these metabolites—including SCFAs, bioactive peptides and transformed polyphenols—are known to activate nutrient-sensing GPCRs, regulate intestinal barrier function, influence bile acid metabolism and modify gut microbiota composition. These processes contribute indirectly to enhanced GLP-1 secretion. These observations support the emerging concept that fermented foods act as multi-component nutritional modulators of the gut–microbiota–L-cell axis rather than through a single biochemical pathway [188,189].

A critical limitation in demonstrating the effects of fermented foods in a reproducible clinical setting is the substantial variability inherent in these food matrices. Products such as kefir, kimchi, tempeh and kombucha can differ greatly in terms of their microbial composition, colony-forming unit (CFU) counts, starter-culture viability, fermentation duration, oxygen exposure and substrate availability. All of these parameters strongly influence the generation of postbiotic metabolites, including SCFAs, organic acids, bioactive peptides and microbial lipid derivatives, which all contribute to incretin-related signalling. Matrix effects, such as fat content, protein structure, buffering capacity and viscosity, modify microbial activity and nutrient release during digestion. Consequently, two products with identical labels may contain markedly different microbial communities and postbiotic profiles, resulting in inconsistent GLP-1 responses across studies and individuals. The absence of standardized production protocols and analytical benchmarks remains a major barrier to achieving consistent incretin outcomes in clinical settings, thereby limiting the development of evidence-based nutritional recommendations for fermented foods.

### 5.4. Fermented Food-Derived Incretin Stimulation in Relation to GLP 1 Receptor Agonist Therapy

The remarkable clinical success of glucagon-like peptide-1 receptor agonists (GLP-1RAs) has transformed the management of obesity, type 2 diabetes, and cardiometabolic disease. This has established GLP-1 signalling as one of the most important therapeutic targets in modern metabolic medicine [191,192]. Consistent results from large cardiovascular outcome trials demonstrate that GLP-1RAs reduce major adverse cardiovascular events, improve glycemic control, and promote sustained weight loss. A comprehensive meta-analysis by Kristensen et al. [193] confirmed significant reductions in cardiovascular and renal events among patients with type 2 diabetes. Sattar et al. (2021) [37] concluded that these agents reduce cardiovascular events, all-cause mortality, and kidney disease across diverse patient populations. More recent evidence continues to strengthen their clinical profile. Galli et al. [39] analyzed data from 99,599 patients and demonstrated excellent cardiovascular safety and favorable tolerability. Meanwhile, Natale et al. (2025) [194] and Stefanou et al. [38] highlighted additional benefits in chronic kidney disease and stroke prevention, respectively. These studies confirm that GLP-1RAs are highly effective receptor-targeted therapies, setting the standard for metabolic efficacy in incretin-based interventions.

Despite their undeniable therapeutic value, GLP-1RAs act by directly activating the GLP-1 receptor. This bypasses the physiological processes that normally regulate endogenous incretin secretion. Analyses indicate that these drugs stimulate insulin secretion, suppress glucagon release, and delay gastric emptying via sustained receptor activation [195]. In contrast, nutritional strategies do not replace endogenous GLP-1 signalling, but rather amplify its physiological regulation through nutrient sensing, microbial metabolism, and enteroendocrine activation. This distinction is particularly relevant because food-derived signals preserve the complex feedback mechanisms linking the intestinal lumen, gut microbiota, enteroendocrine L-cells, and the gut–brain axis [43,44].

Fermented foods are one of the most biologically complex examples of this physiological regulation, as microbial fermentation simultaneously modifies food composition and generates a broad spectrum of bioactive metabolites that can influence enteroendocrine function. Instead of acting as direct GLP-1 receptor agonists, fermented foods promote endogenous incretin secretion via SCFA production, bile acid transformation, the generation of bioactive peptides, the microbial biotransformation of polyphenols, exopolysaccharide synthesis, and the modulation of intestinal microbial ecology [41,42,44]. These complementary mechanisms converge on nutrient-sensing receptors expressed by enteroendocrine L-cells, thereby enhancing physiological GLP-1 secretion while simultaneously supporting intestinal barrier integrity, immune homeostasis, and microbial diversity. Consequently, fermented foods should be regarded as nutritional modulators of the gut–microbiota–L-cell axis at a systems level rather than as single functional ingredients (Table 3). It is important to distinguish between food and nutrients, because foods function as complex biological matrices, whereas nutrients are single, isolated compounds that perform specific physiological functions.

Of all the fermented foods, fermented dairy products have received the most scientific attention because they contain both probiotic microorganisms and fermentation-derived postbiotic metabolites. Greek yoghurt, kefir and other fermented milks, as well as lactic acid bacteria (LAB)-fermented dairy products, provide viable microorganisms alongside organic acids, exopolysaccharides, bioactive peptides and bacterial cell components which remain biologically active after fermentation [213,214]. Dichter (2026) [215] reported that Greek yoghurt promotes butyrate-producing bacterial taxa and improves intestinal barrier integrity, thereby creating favorable conditions for L-cell responsiveness. Previous studies by Heller (2001) [216] and Kok and Hutkins (2018) [217] identified fermented dairy products as a reliable dietary source of health-promoting microorganisms. Meanwhile, Ng et al. (2011) [209] and Mani-López et al. (2014) [218] showed that the viability of probiotics and the postbiotic composition delivered to the distal intestine are influenced by starter cultures and storage conditions. Makino et al. (2016) [219] also showed that exopolysaccharides produced during yoghurt fermentation can modulate epithelial and immune signalling. This provides further evidence of the indirect enhancement of endogenous GLP-1 secretion by fermented dairy products. The broader nutritional benefits described by Papageorgiou et al. (2024) [220] and the metabolomic biomarkers identified by Li et al. (2021) [221] provide further support for the idea that consuming fermented dairy products is associated with favorable metabolic adaptations.

**Figure 4:**
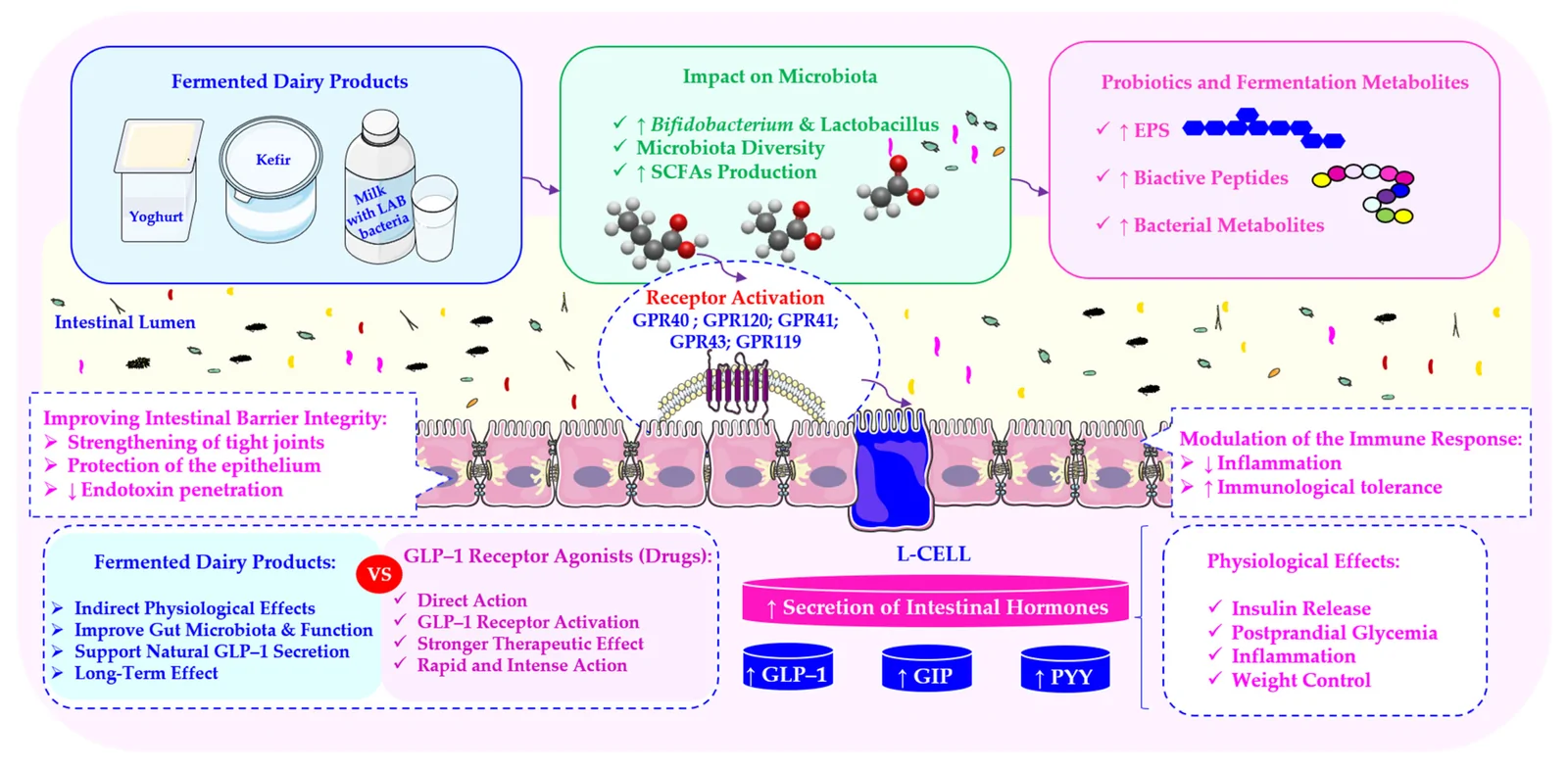
The effect of fermented dairy products on the gut microbiota–enteroendocrine cells (L)–GLP-1 axis and the regulation of glucose homeostasis. Fermented dairy products provide probiotics, postbiotics, and fermentation metabolites that increase the abundance of beneficial gut bacteria, enhance short-chain fatty acid (SCFA) production, strengthen the intestinal barrier, and modulate the immune response. These changes promote the activation of enteroendocrine L cells and increased GLP-1 secretion, leading to improved insulin secretion, glucose tolerance, weight control, and reduced inflammation. Image provided by Servier Medical Art (https://smart.servier.com/), licensed under CC BY 4.0 (https://creativecommons.org/licenses/by/4.0/, accessed on 10 July 2026). Abbreviations: EPS—exopolysaccharides; GLP-1—glucagon-like peptide-1; GIP—glucose-dependent insulinotropic polypeptide; GPR40 (FFAR1)—free fatty acid receptor 1; GPR41 (FFAR3)—free fatty acid receptor 3; GPR43 (FFAR2)—free fatty acid receptor 2; GPR119—G protein-coupled receptor 119; GPR120 (FFAR4)—free fatty acid receptor 4; LAB—lactic acid bacteria, PYY—peptide YY; SCFAs—short-chain fatty acids. Arrows indicate the direction of biological signalling and downstream effects, while upward (↑) and downward (↓) arrows indicate increases and decreases, respectively.

Additional experimental studies reinforce this concept. For example, Fallah et al. (2018) [208] demonstrated that fermented camel milk improves glucose metabolism and inflammatory markers in adolescents with metabolic syndrome. Similarly, Shirouchi et al. (2016) [222] found that *Lactobacillus gasseri*-fermented milk improved glucose tolerance and reduced body weight gain in rodents, and Niibo et al. (2019) [223] reported enhanced insulin secretion in diabetic models. Further evidence from Wu et al. (2021) [224] suggests that microbial interventions can alter the composition of the gut microbiome. Satish Kumar et al. (2022) [225], meanwhile, emphasized the broader role of probiotics in gut–brain and gut–immune communication. While few studies have directly quantified GLP-1 secretion following consumption of fermented foods, accumulated evidence consistently demonstrates improvements in microbial ecology, intestinal barrier function, inflammatory status, and metabolic regulation. These factors are important determinants of endogenous incretin responsiveness (Figure 4).

Therefore, fermented foods and postbiotics are not nutritional equivalents of GLP-1 receptor agonists. Instead, they act as complementary physiological modulators that enhance endogenous incretin activity prior to receptor activation. GLP-1RAs produce predictable effects by directly stimulating the receptors, whereas fermented foods gradually improve the intestinal environment by reshaping microbial metabolism, strengthening epithelial integrity, and generating bioactive metabolites that activate enteroendocrine signalling [41,42,43]. This distinction is central to precision nutrition: fermented foods function as a long-term strategy that supports intrinsic incretin physiology rather than replacing it pharmacologically. Their value lies in optimizing the gut–microbiota–L-cell axis through multiple interconnected pathways that contribute to metabolic improvement rather than in reproducing the magnitude of GLP-1RA therapy.

### 5.5. Protein-Derived Peptides, Amino Acids and GPR119-Dependent Nutrient Sensing

Protein-derived peptides and signalling amino acids activate nutrient-sensing pathways similar to those engaged by fermented foods and postbiotics, with their metabolites converging on GPR119 to reinforce fermentation-driven incretin signalling [226,227], while protein digestion generates bioactive compounds that modulate enteroendocrine function and link protein intake to endogenous GLP-1 regulation [228,229]. Studies have established the structural basis of GPR119 activation relevant to nutrient-derived metabolites. GPR119 is a class A G protein-coupled receptor that is predominantly expressed in pancreatic β-cells and intestinal enteroendocrine L-cells. There, it contributes to glucose homeostasis, insulin secretion, and feeding behavior [40]. Cryo-electron microscopy structures of the GPR119–Gs complex demonstrate that activation of the receptor depends on ligand-induced conformational changes within transmembrane domain 5. This forms a hydrophobic cavity that can accommodate endogenous monounsaturated lipid amides. These lipid derivatives, including oleoylethanolamide (OEA), are the primary physiological agonists of GPR119. Their binding promotes efficient coupling to Gs proteins and increases intracellular cAMP, a key signalling event that drives GLP-1 and GIP secretion [40].

Dietary proteins and peptides do not activate GPR119 directly. Instead, they indirectly influence this pathway by modifying the luminal and microbial environment in a way that favors the production of endogenous lipid amides. Through their effects on microbial fermentation, epithelial metabolism, and nutrient-driven lipid processing, protein-derived metabolites enhance the availability of natural GPR119 agonists, rather than acting as ligands themselves. This places proteins within the broader nutrient-sensing network that regulates incretin secretion.

Whey-derived peptides have emerged as important modulators of metabolic signalling pathways involved in glucose regulation and gut hormone secretion [229,230,231]. Whey proteins comprise several biologically active fractions, including β-lactoglobulin, α-lactalbumin, immunoglobulins, bovine serum albumin, proteose-peptone fractions and glycomacropeptide. Digestion or food processing generates bioactive peptides from whey proteins that have diverse physiological properties [43,232]. These peptides exhibit antioxidant, antihypertensive, antimicrobial and immunomodulatory activities, and their biological effects are determined by their sequence, size, hydrophobicity and structural conformation [233]. Beyond these functions, several peptide fractions enhance enteroendocrine signalling by stimulating GLP-1 secretion or modifying epithelial responsiveness to luminal nutrients. While whey-derived peptides do not directly bind to GPR119, they can influence microbial fermentation and epithelial nutrient sensing, thereby increasing the generation of endogenous lipid agonists and supporting GPR119-dependent cAMP signalling [104].

The gut microbiota plays a crucial role in linking dietary protein metabolism to GPR119 activation. Microbial fermentation of proteins produces metabolites including endocannabinoid-like compounds, *N*-acylethanolamines (NAEs), bioactive lipids and phenolic derivatives [234,235]. Monounsaturated lipid derivatives and NAEs, particularly oleoylethanolamide, are well-established endogenous GPR119 ligands [42,236,237]. Activation of GPR119 by these compounds enhances GLP-1 secretion and modulates insulin sensitivity, energy balance, and inflammatory signalling [238]. Consequently, dietary proteins indirectly contribute to incretin regulation by shaping microbial metabolic activity and promoting the generation of endogenous GPR119 agonists rather than functioning as direct receptor ligands [238,239].

As shown in reference [18], enteroendocrine L-cells integrate signals from multiple nutrient-sensing receptors to generate coordinated intracellular responses that enhance incretin secretion [18,240]. Protein-derived nutrients activate a network involving GPR119, GPR40, the calcium-sensing receptor, PEPT1 and sodium-coupled amino-acid transporters, and simultaneous stimulation of GPR119 and GPR40 produces stronger GLP-1 release [241], while amino acids such as glutamine further increase L-cell responsiveness when combined with GPR119 activation [242]. In parallel, microbial protein fermentation supplies lipid-like metabolites that activate GPR119, and peptide-derived signals support epithelial and immune function, with studies in experimental models and humans confirming that GPR119 activation reduces food intake, improves glucose tolerance and increases endogenous incretin release [40,41]. These findings show that protein-derived peptides, glutamine and signalling amino acids regulate endogenous GLP-1 by modifying nutrient-sensing pathways and shaping the microbial metabolic environment that favors GPR119 activation, supporting physiological incretin secretion through complementary mechanisms rather than acting as direct pharmacological agonists.

### 5.6. Polyphenols and Plant Bioactives and Its Effect on GLP-1

Building on the dietary compounds discussed in previous sections, polyphenols and other plant-derived bioactives are an additional class of nutrients that modulate endogenous GLP-1 secretion [23,243]. Unlike the effects of fermentable fibers, which are primarily mediated through the production of SCFAs by microbes, the effects of polyphenols on incretin physiology are influenced by direct interactions with enteroendocrine cells, microbial biotransformation into bioactive metabolites and modulation of enzymes involved in GLP-1 signalling. These complementary mechanisms considerably broaden the nutritional regulation of the enteroendocrine system [244,245].

Experimental studies have demonstrated that the polyphenols found in green tea, turmeric, berries, grapes and coffee interact with the intestinal epithelium and stimulate enteroendocrine L-cells via multiple nutrient-sensing pathways [245,246]. Experiments using human NCI-H716 cells, as well as murine GLUTag and STC-1 cell lines, revealed that extracts of both digested and undigested green tea and turmeric stimulate GLP-1 secretion. This indicates that these compounds remain biologically active at the luminal surface prior to systemic absorption [247,248]. Coffee polyphenol extract increases active GLP-1(7-36) amide concentrations via a cAMP-dependent mechanism that is independent of GPR119. This demonstrates that plant bioactives can activate incretin secretion through signalling pathways that are distinct from the classical lipid-sensitive receptor system [249]. Curcumin enhances GLP-1 secretion via Ca^2+^/calmodulin-dependent protein kinase II signalling and redox-sensitive pathways [250,251]. In contrast, ellagitannins isolated from black raspberry seeds regulate GLP-1 secretion through bitter taste receptor signalling and structure-dependent receptor interactions [252]. These findings suggest that polyphenols can directly activate enteroendocrine nutrient-sensing mechanisms, independent of SCFA-mediated signalling.

A second mechanism involves the extensive microbial metabolism of dietary polyphenols. As many native polyphenols are only partially absorbed in the small intestine, significant quantities reach the colon. There, they are converted by gut microbiota into aglycones, phenolic acids, urolithins and various low-molecular-weight metabolites with increased biological activity [253,254,255,256]. These microbial metabolites often have greater bioavailability and biological potency than their parent compounds, allowing for more effective interaction with enteroendocrine cells. Fermented berry beverages produce phenolic metabolites that can modulate DPP-IV activity, thereby influencing GLP-1 availability [257]. Similarly, the metabolic effects of yerba maté depend partly on the microbial conversion of ferulic acid derivatives [258]. Fermented tea is another interesting example, producing teadenol A—a novel GPR120 agonist that stimulates GLP-1 secretion [259]. These observations emphasize that microbial metabolism substantially amplifies and diversifies the incretin-regulating potential of dietary polyphenols.

**Figure 5:**
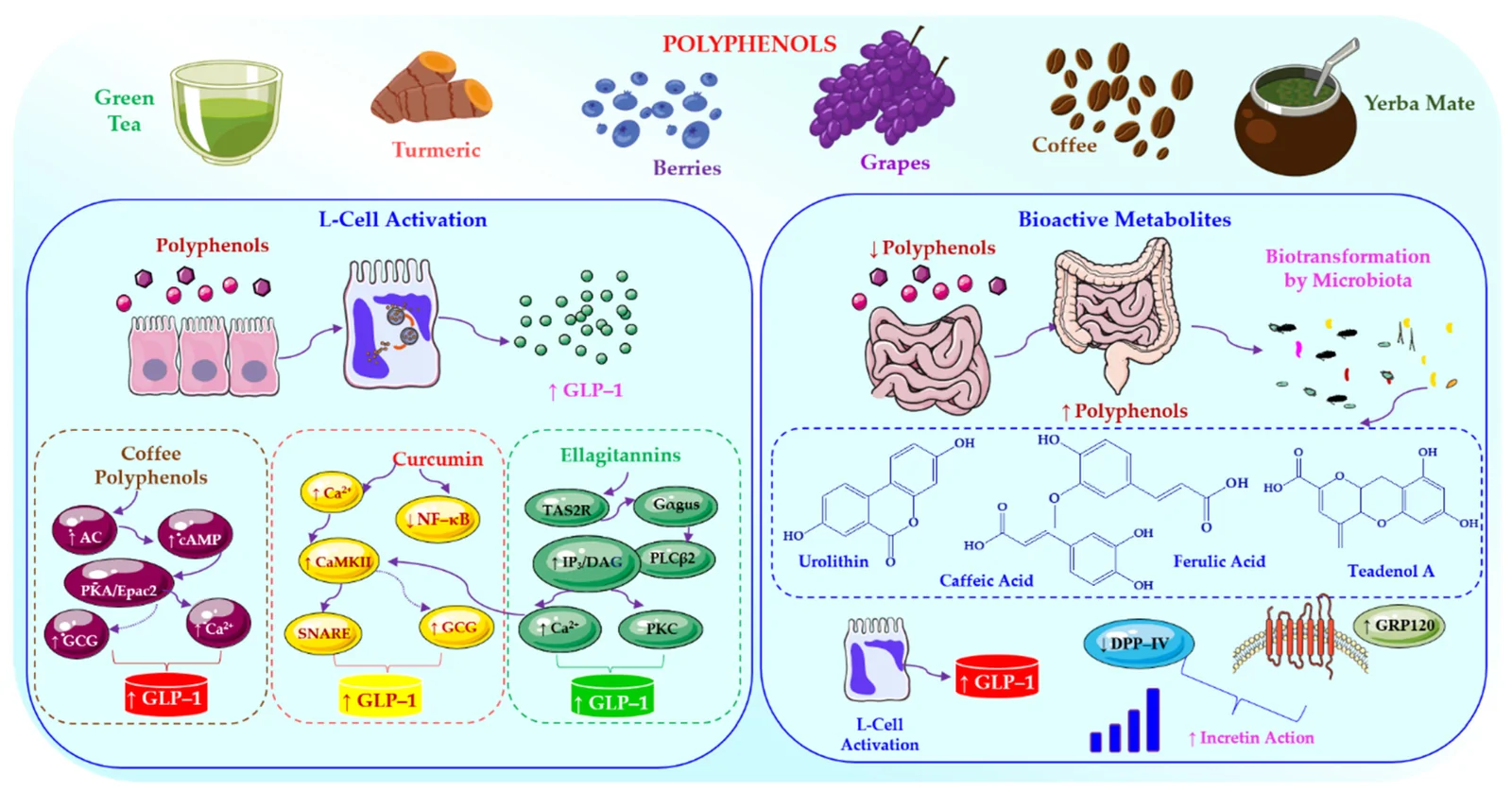
The effect of microbially derived polyphenols and their metabolites on GLP-1 secretion and the regulation of glucose homeostasis. Polyphenols in foods, including those in green tea, turmeric, berries, grapes, coffee, and yerba mate, can increase GLP-1 secretion through two complementary mechanisms. The first involves direct activation of enteroendocrine L cells via signaling pathways dependent on cAMP, Ca^2+^/CaMKII, and bitter taste receptors. The second involves the gut microbiota biotransforming polyphenols into bioactive metabolites that enhance GLP-1 secretion, inhibit DPP-IV activity, and modulate receptor activity, such as GPR120. Image provided by Servier Medical Art (https://smart.servier.com/), licensed under CC BY 4.0 (https://creativecommons.org/licenses/by/4.0/, accessed on 10 July 2026). Abbreviations: AC—adenylyl cyclase; CaMKII—calmodulin-dependent protein kinase II; cAMP—cyclic adenosine 3′,5′-monophosphate; DPP-IV—dipeptidyl peptidase IV (CD26); Epac2—exchange protein directly activated by cAMP 2; Gαgust—gustducin alpha subunit; GCG—glucagon gene; GLP-1—glucagon-like peptide-1; GPR120 (FFAR4)—free fatty acid receptor 4; IP_3_—inositol 1,4,5-trisphosphate; NF-κB—nuclear factor kappa B; PKA—protein kinase A; PKC—protein kinase C; PLCβ2—phospholipase C beta 2; SNARE—soluble *N*-ethylmaleimide-sensitive factor attachment protein receptor; TAS2R—taste receptor type 2. Arrows indicate the direction of the proposed biological and intracellular signalling pathways. Upward arrows (↑) indicate increased activity or secretion, whereas downward arrows (↓) indicate decreased activity or inhibition.

Polyphenols not only stimulate GLP-1 secretion but also strengthen incretin activity by limiting enzymatic degradation. Many flavonoids and related phytochemicals inhibit dipeptidyl peptidase-4 (DPP-4), thereby prolonging the biological half-life of circulating GLP-1 [260]. The glucose-lowering activity of myricetin is significantly reduced when it is administered alongside lectin-rich proteins, suggesting that the preservation of GLP-1 through DPP-IV inhibition is one of its primary mechanisms of action [261]. Curcumin and its Ru(II)-based derivatives also inhibit DPP-IV and improve insulin sensitivity [262]. Additionally, grape seed procyanidins regulate the DPP-4/Sirt1 pathway, linking incretin preservation to enhanced cellular resilience and metabolic homeostasis [263]. Fermented berry phenolics exert similar effects by modulating both DPP-IV activity and GLP-1 substrate dynamics [257]. Thus, polyphenols influence incretin biology at two complementary levels, simultaneously enhancing GLP-1 secretion and reducing its degradation (Figure 5).

The physiological consequences of polyphenol-mediated GLP-1 signalling extend far beyond glucose homeostasis. Black soybean seed coat polyphenols have been shown to increase intracellular cAMP, stimulate GLP-1 secretion and promote endothelial nitric oxide synthase (eNOS) phosphorylation. This links enteroendocrine activation with improved vascular endothelial function [264,265]. Cacao polyphenols influence circadian clock gene expression via GLP-1-dependent pathways [266], whereas chlorogenic acid exhibits neuroprotective activity in experimental models of Parkinson’s disease by acting as a GLP-1 secretagogue [267]. Curcumin also modulates the microbiota–bile acid–TGR5/FXR axis, which partially compensates for impaired GLP-1 signalling [268]. Turmeric-based beverages have been shown to improve postprandial glycemia and satiety in human intervention studies [269]. These findings demonstrate that modulation of endogenous GLP-1 is one component of a broader network through which polyphenols influence cardiovascular, neurological, circadian and metabolic physiology (Table 4).

Table 5 summarizes the main ways in which polyphenols and plant-derived bioactive compounds increase the secretion of endogenous GLP-1 and influence incretin-related signalling.

Thus, current evidence suggests that polyphenols and plant-derived bioactives regulate endogenous GLP-1 activity via several complementary mechanisms. These include the direct stimulation of enteroendocrine L-cells, the microbial biotransformation of these cells into highly active metabolites, the inhibition of DPP-IV and the activation of receptors such as GPR120 [260]. Unlike the pharmacological GLP-1 receptor agonists semaglutide, liraglutide, dulaglutide and tirzepatide, which produce therapeutic effects by directly activating the receptor, dietary polyphenols enhance endogenous incretin physiology before the receptor is activated by modifying nutrient sensing, epithelial signalling and gut microbial metabolism. Consequently, polyphenols should be regarded as complementary nutritional modulators of the incretin axis, rather than natural substitutes for GLP-1 receptor agonists. They have the greatest therapeutic potential when it comes to supporting physiological GLP-1 secretion within an integrated diet–microbiota–host signalling network [243,245]. This provides a foundation for future precision nutrition strategies aimed at improving metabolic health.

### 5.7. Omega-3 Fatty Acids, GPR120 Activation and Anti-Inflammatory Signalling

The GLP-1-enhancing actions of polyphenols, which are mediated through microbial biotransformation and improve epithelial integrity while reducing intestinal inflammation, provide a natural transition to the role of omega-3 fatty acids. While these two dietary compound classes act through distinct molecular mechanisms, they both converge on pathways that promote endogenous GLP-1 secretion and improve metabolic homeostasis [278,279]. In particular, eicosapentaenoic acid (EPA) and docosahexaenoic acid (DHA), which are found in high concentrations in marine fish and seafood, act as endogenous ligands for G protein-coupled receptor 120 (GPR120), also known as free fatty acid receptor 4 (FFAR4). This lipid-sensing receptor is expressed in adipocytes, macrophages, intestinal epithelial cells, and enteroendocrine L-cells [101,280]. Upon binding to EPA or DHA, GPR120 predominantly activates β-arrestin-dependent signalling, which inhibits transforming growth factor-β-activated kinase 1 (TAK1). This subsequently suppresses nuclear factor-κB (NF-κB) and c-Jun *N*-terminal kinase (JNK) signalling. This reduces Toll-like receptor 4 (TLR4)-mediated production of pro-inflammatory cytokines, including tumor necrosis factor-α (TNF-α), which is a major contributor to obesity-associated insulin resistance [281]. Oh and Walenta (2014) [282] demonstrated that activation of GPR120 functions as a physiological anti-inflammatory switch in macrophages. Conversely, Talukdar et al. (2011) [100] showed that fatty acid-sensing GPCRs, including GPR120, improve insulin sensitivity by suppressing chronic metabolic inflammation. These anti-inflammatory actions create a physiological environment that favors normal enteroendocrine function and supports endogenous GLP-1 secretion.

The intestinal component of this signalling axis is important because GPR120 is highly expressed in enteroendocrine L-cells. Here, EPA and DHA stimulate the mobilization of intracellular calcium through Gq-dependent signalling, thereby promoting the secretion of GLP-1 [283]. Cheshmehkani et al. [284] demonstrated that diets enriched with fish oil significantly increased colonic FFAR4 expression while reducing TNF-α levels simultaneously, indicating that omega-3 fatty acids improve both receptor availability and the intestinal environment required for efficient incretin release. Omega-3 fatty acids support incretin physiology by preserving epithelial integrity and reducing local inflammatory stress, both of which influence nutrient sensing and hormone secretion.

Consistent with these findings, Anbazhagan et al. [285] reported that GPR120 activation strengthens epithelial barrier function and reduces intestinal inflammatory signalling. Conversely, Miccadei et al. [286] emphasized the broader immunomodulatory role of omega-3 polyunsaturated fatty acids (PUFAs) in maintaining intestinal immune homeostasis. These studies demonstrate that omega-3 fatty acids support GLP-1 biology by directly activating receptors and optimizing the intestinal microenvironment.

Further clinical studies support the metabolic relevance of this signalling pathway. In the EPICO randomized clinical trial, for example, marine omega-3 supplementation was found to enhance FFAR4 activation in peripheral blood mononuclear cells (PBMCs) and reduce systemic inflammatory markers in obese individuals [287]. Senatorov and Moniri [288] reviewed the biology of FFAR4 across multiple human tissues and concluded that activation of the receptor consistently suppresses inflammatory signalling, irrespective of cell type, thereby highlighting its systemic importance. Similarly, Chitre et al. [289] described GPR120 as a promising therapeutic target in neuroinflammatory disorders, and Carullo et al. [101] discussed its potential as a target for novel type 2 diabetes therapies due to its insulin-sensitizing and anti-inflammatory properties. More recently still, Teyani and Moniri [290] demonstrated that GPR120 exhibits biased agonism, whereby different ligands preferentially activate distinct intracellular signalling pathways. This observation is particularly relevant because naturally occurring EPA and DHA appear to induce balanced receptor activation, supporting anti-inflammatory signalling, metabolic regulation, and endogenous GLP-1 secretion simultaneously.

Unlike pharmacological GLP-1 receptor agonists, which activate the GLP-1 receptor directly, omega-3 fatty acids act upstream by modulating nutrient sensing, inflammatory signalling, and the responsiveness of enteroendocrine cells. FFAR4 is expressed in several metabolically active tissues, including adipose tissue, macrophages, intestinal epithelial cells and L-cells. This allows EPA and DHA to coordinate metabolic responses across multiple organs [290]. Consequently, dietary PUFAs simultaneously improve insulin sensitivity, reduce chronic inflammation, preserve epithelial barrier integrity and facilitate physiological GLP-1 secretion. Rather than replacing pharmacological GLP-1 receptor agonists, omega-3 fatty acids should be regarded as complementary nutritional modulators that enhance endogenous incretin function through multi-organ physiological mechanisms. This concept is consistent with the existing evidence that supports the therapeutic potential of omega-3 fatty acids in the treatment of metabolic, inflammatory and neurodegenerative disorders [286,288,289]. Table 6 summarizes the diverse molecular mechanisms through which omega-3 fatty acids influence GLP-1 physiology.

Thus, the data suggests that omega-3 fatty acids support endogenous incretin physiology via coordinated mechanisms in the intestine, immune system and adipose tissue, rather than through a single signalling pathway. Therefore, marine EPA and DHA function as nutritional modulators of the gut–pancreas axis, helping to maintain cardiometabolic health without replicating the direct receptor agonism characteristic of GLP-1-based pharmacotherapy.

## 6. Inter-Individual Variability and Precision Nutrition: Why Responses Differ Between Individuals

Consistent evidence from human and animal studies shows that endogenous GLP-1 regulates satiety, gastric emptying, glycemic control and body-weight dynamics via conserved physiological pathways [1,2,3,4,5,6]. Additional observations from gastric-bypass models and GLP-1RA trials confirm the metabolic relevance of incretin signalling [7,8,9,10]. Dietary interventions further demonstrate that fibers, polyphenols, protein hydrolysates and fermented foods stimulate endogenous GLP-1 via SCFA production, nutrient-sensing receptors and microbiota-dependent mechanisms [122,152,154,156,158,159,172,175,177,183,196,198,291], forming convergent metabolic pathways involving gastric emptying, glucose-dependent insulin secretion, FFAR2/3, GPR119, FFAR4 and TGR5 signalling [1,3,5,11,47,77,86]. These pathways support long-term metabolic health by modulating the gut–microbiota–enteroendocrine axis in the long term [19,23,45,167,292].

Responses to endogenous GLP-1 vary among individuals due to differences in gastrointestinal physiology, genetics, microbiota composition, and metabolic state. Studies have shown substantial variation in L-cell nutrient sensing, Ca^2+^ signalling, epithelial function, and autonomic regulation [22,52,53,91,92,97]. Microbiota-dependent factors strongly influence incretin secretion, as enterotype-specific fermentation, short-chain fatty acid (SCFA) production and bile acid metabolism generate distinct GLP-1 responses to fiber, polyphenols and fermented foods [19,45,46,47,48,49,77,80,177,183,190]. Furthermore, host genetics and metabolic phenotype modulate the abundance of receptors, the affinity of ligands and intracellular signalling across FFAR4, FFAR2/3, GPR119 and TGR5, thereby influencing incretin release and glucose homeostasis [16,52,55,86,92,97,103,108,112,114,115].

Table 7 summarizes the main biological factors responsible for the variability in how individuals respond to endogenous GLP-1 and shows how these factors can affect the effectiveness of dietary strategies designed to increase incretin secretion.

Recent systems biology data show that integrating microbiome composition, metabolomic profiles, genetics and habitual diet enables more accurate prediction of GLP-1 responsiveness [293], as microbial metabolites interact with FFAR2/3, GPR119, FFAR4 and TGR5 to generate signatures tightly linked to incretin secretion [48,50,51], with enterotype-specific fermentation shaping responses to fiber, polyphenols and fermented foods [45,46,177] and metabolomic profiling of endogenous GPR119/FFAR4 ligands further refining these predictions [111,114,119]. Biological sex and cyclical hormonal variation also modulate GLP-1 release, as estrogen-responsive L-cell signalling and fluctuations in estradiol and progesterone alter gastric emptying, vagal activity, bile-acid metabolism and nutrient sensing [54], supporting precision-nutrition strategies that align dietary composition with microbiome, receptor profile, metabolic phenotype and hormonal state to optimise endogenous GLP-1 and long-term cardiometabolic health.

## 7. Clinical Integration of Nutritional GLP-1 Modulation

Findings suggest that enhancing endogenous GLP-1 through diet should complement, rather than replace, pharmacological therapy. Combining nutritional strategies with GLP-1 receptor agonists (GLP-1RAs) improves metabolic outcomes in obesity, type 2 diabetes and metabolic syndrome [15,294,295]. Clinical trials confirm that GLP-1RAs reduce appetite, improve glycemia and support weight loss [8,9,10], while dietary components such as fermentable fibers, polyphenols, protein hydrolysates and fermented foods stimulate endogenous GLP-1 through gastric emptying, nutrient sensing and microbiota-dependent pathways [23,152,158,198]. This provides additional cardiometabolic benefits, which are supported by outcome trials [37,39,193].

This combined rationale reflects the shared pathophysiological mechanisms of metabolic disorders. Dietary GLP-1 enhancement can improve epithelial integrity, inflammation, SCFA production, bile acid signalling and L-cell activation, while GLP-1RAs reinforce these processes through direct receptor activation. Further study is required to identify reliable predictors of treatment response, such as SCFA profiles, bile acid signatures, receptor expression and metabolomic fingerprints [46,50,51], including analyses of L-cell heterogeneity, receptor polymorphisms and host–microbiome interactions [52,53,55], as well as investigations into neural–enteroendocrine communication, ER–mitochondria signalling and sex-specific nutrient sensing [52,54,55]. Table 8 summarizes the therapeutic value of diets that increase endogenous GLP-1.

Thus, precision nutrition strategies that modulate endogenous GLP-1 may enhance the effectiveness of pharmacological incretin therapy, although responses to such diets vary due to genetics, metabolic phenotype and microbiome composition; integrating multi-omics biomarkers with detailed dietary assessments could enable targeted interventions that optimize endogenous and pharmacological GLP-1 activity.

## 8. Limitations and Future Research

Current data suggests that nutrition can increase the secretion of endogenous GLP-1, but there are still several limitations. Most studies rely on short-term interventions, which limits the ability to draw conclusions about long-term metabolic effects [296,297]. Additionally, the use of heterogeneous dietary protocols, fiber sources, and bioactive food matrices hinders comparability and standardization [3,296]. Additional variability arises from differences in age, metabolic status, obesity severity and medication use [297,298], while many mechanistic insights originate from animal or in vitro models that differ substantially from human physiology [3,299]. Key interactions between the epithelial barrier, the immune system, microbial metabolism and neural-enteroendocrine signalling via G-protein-coupled receptors (FFAR2/3, FFAR4, GPR119 and TGR5) are not well characterized in vivo [46,77,296], and differences in the composition of the microbiome, genetics and metabolic phenotype complicate interpretation further [293]. A major challenge is the absence of reliable biomarkers of GLP-1 responsiveness, as circulating GLP-1 levels may not reflect local enteroendocrine activity or downstream signalling, underscoring the need for methodological harmonization [3,297,299].

Future research should prioritize long-term, well-controlled clinical trials that integrate detailed phenotyping with microbiome sequencing, metabolomics, bile acid profiling and nutrient-sensing receptor characterization [300,301,302,303,304]. Multi-omics approaches may clarify how diet, microbial ecology and host metabolism interact to regulate incretin physiology, enabling the development of precision nutrition frameworks that predict GLP-1 responsiveness based on enterotype, SCFA-producing capacity, epithelial barrier status, receptor expression and metabolic phenotype [46,304]. Systems biology models linking microbial fermentation dynamics with host GPCR signalling (FFAR2/3, GPR119, FFAR4 and TGR5) could refine our mechanistic understanding. Rather than isolating single nutrients, future studies should examine the combined effects of fibers, peptides, polyphenols, fermented foods, postbiotics and omega-3 fatty acids [23,189]. They should also assess how dietary interventions interact with GLP-1 receptor agonists in clinical practice, considering the impact on adherence, dosage optimization and cardiometabolic outcomes [305,306,307,308]. Integrating nutritional science with pharmacology, microbiome research and precision medicine will be essential for developing personalized dietary strategies that complement GLP-1-based therapies [295].

## 9. Conclusions

This review shows that changing what we eat to boost the body’s own GLP-1 production is a good way to support metabolic health. Nutritional factors influence incretin physiology via various upstream pathways, including microbial fermentation, postbiotic metabolites, bioactive lipids, bile acid signalling, amino acid sensing, and peptide-driven epithelial activation. Together, these pathways shape the physiological environment in which endogenous GLP-1 is produced and integrated into metabolic regulation.

A key finding is the pivotal role of the gut microbiota in linking dietary intake with incretin responses. For example, microbial metabolites such as propionate and butyrate stimulate GLP-1 release via FFAR2/3, while also strengthening epithelial integrity, modulating immune tone, and influencing neural communication. Microbial biotransformation of polyphenols generates bioactive metabolites that activate L-cells, while fermented foods provide additional microbial signals that reinforce nutrient sensing. Lipid-derived metabolites and *N*-acylethanolamines engage GPR119; omega-3 fatty acids activate FFAR4/GPR120; and protein-derived peptides interact with nutrient-sensing pathways that amplify endogenous incretin responses. These findings emphasize that the dietary regulation of GLP-1 arises from the interconnected interactions of the microbiota, epithelium, immune system and endocrine system.

This review emphasizes the importance of viewing nutritional and pharmacological strategies as complementary. GLP-1 receptor agonists are the most potent and predictable way of enhancing incretin signalling through direct receptor activation, and are therefore central to the treatment of obesity and type 2 diabetes. While dietary interventions are physiologically meaningful, they have a more modest effect than pharmacological GLP-1 receptor activation. They are valuable in improving the metabolic and intestinal environment in which endogenous incretin signalling occurs by promoting microbial fermentation, supporting epithelial function, regulating bile acid metabolism, and engaging nutrient-sensing pathways. This complementarity suggests that evidence-based dietary strategies may augment, stabilize or prolong the benefits of GLP-1-based pharmacotherapy, particularly in individuals with impaired endogenous secretion or gut microbial dysbiosis.

The studies also highlight significant inter-individual variability in responsiveness to GLP-1-enhancing dietary strategies. Differences in microbiome composition, fermentation capacity, receptor expression, bile acid metabolism, epithelial integrity and host metabolic phenotype all contribute to these heterogeneous outcomes. These observations support a shift from population-level dietary recommendations towards precision nutrition approaches that integrate microbiome profiling, metabolomics, receptor biology and dietary habits to predict individual responsiveness.

When considered as a whole, the findings of this review provide a framework for understanding how endogenous GLP-1 regulation arises from the coordinated interactions between diet, gut microbiota, nutrient-sensing pathways, and host metabolism. The continued integration of nutritional science, microbiome research, molecular endocrinology, and clinical medicine is essential to develop personalized dietary interventions that can complement pharmacological therapies and improve the prevention and management of obesity, type 2 diabetes, and related cardiometabolic disorders.

## Figures and Tables

**Table 1:** Effects of fermentable fibers and resistant starch on microbial fermentation, enteroendocrine signalling and metabolic outcomes.

Study Type	Model System	Principal Fermentation Products	Main Nutrient-Sensing Pathway	Effect on Enteroendocrine Hormone Secretion	Preclinical Outcomes	Clinical Outcomes	References
Rodent resistant starch fermentation studies	Rodent	Increased acetate, propionate and butyrate	SCFA activation of FFAR2/FFAR3	Sustained increase in GLP-1 and PYY	Reduced body fat, increased satiety, improved glycemic control	—	[123]
Rodent resistant starch metabolic review	Rodent	Increased SCFA (mixed profile)	Fermentation-dependent stimulation of endogenous GLP-1	Increased GLP-1 contributing to improved insulin sensitivity	Reduced adiposity, improved insulin sensitivity	—	[126]
Human barley kernel intervention	Human	Increased SCFA	Sustained microbial fermentation	Increased GLP-1 on the following day	—	Increased satiety, improved glycemic control	[127]
Human barley indigestible carbohydrate intervention	Human	Increased SCFA	Fermentation-dependent incretin stimulation	Increased GLP-1	—	Reduced food intake	[128]
Portal metabolite flux study in pigs	Pig	Increased butyrate absorption	Portal SCFA signalling	Modulation of GLP-1	Improved postprandial glucose and insulin responses	—	[132]
Human inulin intervention	Human	Acute SCFA increase	SCFA production following colonic fermentation	No acute GLP-1/PYY change; possible ghrelin reduction	—	Trend towards reduced appetite	[133]
Human fermentable fibre intervention	Human	Increased SCFA	Activation of FFAR2/FFAR3	Modest GLP-1 increase	—	Increased satiety	[134]
Human AXOS and resistant starch intervention	Human	Increased microbial fermentation	FFAR2/FFAR3-mediated incretin signalling	Increased incretin response	—	Improved overnight glucose tolerance	[135]
Rodent resistant starch–POMC study	Rodent	Increased SCFA	SCFA-mediated GLP-1/PYY secretion with hypothalamic signalling	Increased GLP-1/PYY with increased hypothalamic POMC	Reduced body fat	—	[136]
Rodent enzymatically synthesised glycogen study	Rodent	Increased cecal SCFA	Fermentation-induced L-cell activation	Increased cecal GLP-1	Reduced food intake	—	[137]
Human cereal fibre intervention	Human	Increased microbial fermentation	Modulation of enteroendocrine secretion	Altered GLP-1 and GIP	—	Improved glucose tolerance	[138]
Pig SCFA hormone study	Pig	Increased acetate	SCFA-mediated appetite hormone regulation	Modulation of appetite hormones	Reduced feed intake, reduced fat deposition	—	[139]
Sow fibre fermentation study	Sow	Increased SCFA	Fermentation-induced satiety signalling	Enhanced endocrine satiety response	Reduced physiological stress, improved metabolic profile	—	[140]
Human evening fibre intervention	Human	Increased overnight SCFA	Sustained microbial fermentation	Increased GLP-1 on the following day	—	Improved glucose tolerance	[141]
Review of soluble dietary fibres	Review	Increased SCFA	Increased intestinal viscosity and microbial fermentation activating FFAR2/FFAR3	Summary of GLP-1/PYY evidence	Summary of metabolic effects in animal models	Summary of glycemic effects in human studies	[142]

Abbreviations: AXOS—arabinoxylan oligosaccharides; FFAR2/3—free fatty acid receptors 2 and 3; GIP—glucose-dependent insulinotropic polypeptide; GLP-1—glucagon-like peptide-1; GLP-2—glucagon-like peptide-2; POMC—proopiomelanocortin; PYY—peptide YY; RS, resistant starch; SCFA—short-chain fatty acids; — not reported or not applicable.

**Table 2:** Food-derived modulators of endogenous GLP-1 secretion: mechanisms of action, metabolic effects and translational relevance.

Dietary Product	Model System	Principal Bio-Active Components	Primary Mechanism Enhancing Endogenous GLP-1	Effect on Enteroendocrine Hormone Secretion	Preclinical Outcomes	Clinical Outcomes	Level of Evidence	References
Chicory root, oligofructose	Human + animal	Inulin-type fructans	Colonic fermentation → SCFA production → FFAR2/FFAR3 activation	Increased GLP-1, PYY; microbiota modulation	Reduced adiposity; improved appetite regulation (animal)	Reduced body weight, fat mass, waist circumference; improved glucose metabolism (human)	Human RCTs, meta-analysis, animal studies	[122,143,163]
Jerusalem artichoke	Human + animal	Inulin-type fructans	SCFA production; reduced GIP; improved insulin signalling	Increased GLP-1	Improved insulin sensitivity, β-cell function (animal)	Reduced postprandial glucose and GIP; improved insulin sensitivity (human)	Human + animal studies	[144,164,165]
Garlic	Cell + animal	Allicin; GaELNs	TRPA1 activation; microbiota modulation	Preserved gut hormone homeostasis	Improved glycemic control; protection against HFD-induced dysregulation	—	Animal and cell-based studies	[166,167]
Onion	Experimental (preclinical)	Polyphenols; organosulfur compounds	Anti-inflammatory signalling; modulation of incretin pathways	Improved oxidative and vascular markers	Improved metabolic profile	—	Experimental studies	[154,168]
Apple-derived ingredients	Experimental (preclinical)	Polyphenols	Regulation of inflammatory and metabolic signalling	Improved metabolic biomarkers	Improved vascular and metabolic profile	—	Experimental studies	[154]
Polyphenol-rich curry	Human clinical trial	Polyphenols; spice bio-actives	Direct stimulation of L-cell nutrient sensing	Increased GLP-1	—	Dose-dependent GLP-1 increase; improved glucose homeostasis	Human clinical trial	[152,153]
Chilled potatoes	Human cross-over	Resistant starch	Delayed digestion; microbial fermentation; SCFA production	Modulation of incretin secretion	—	Reduced postprandial glucose, insulin, GIP	Human cross-over trials	[146]
Cooked-and-cooled rice with potatoes	Human intervention	Resistant starch	Increased fermentable starch delivery; enhanced incretin signalling	Improved incretin response	—	Improved glycemic and insulinemic responses	Human intervention study	[169]
Green banana flour	Animal	Resistant starch; dietary fiber	Microbiota modulation; SCFA production	Altered GLP-1 and appetite hormones	Improved glucose and lipid metabolism; increased GLP-1; reduced adiposity	—	Animal studies	[151,170]
Sweet potato leaves	Experimental (preclinical)	Caffeoylquinic acids; polyphenols	Increased GLP-1; modulation of DPP-IV	Improved endocrine profile	Reduced hyperglycemia; improved lipid profile	—	Experimental studies	[156,171]
Rice protein hydrolysates	Cell + animal	Bioactive peptides	Direct GLP-1 stimulation; DPP-IV inhibition	Increased circulating GLP-1	Reduced postprandial glycemia	—	Cell + animal studies	[156]
Wheat protein hydrolysate	Cell + animal	Low-molecular-weight peptides	Ca^2+^/calmodulin-dependent kinase II signalling via GPRC6A	Enhanced GLP-1	Improved glucose tolerance	—	Cell + animal studies	[159]
Oat bran and oat β-glucan	Human RCTs + meta-analyses	β-Glucan	Increased viscosity; delayed gastric emptying; SCFA production	Increased GLP-1 and satiety	—	Improved glycemic control; improved insulin sensitivity	Human RCTs, meta-analyses	[172,173,174,175,176]

Abbreviations: AMPK—AMP-activated protein kinase; DPP-IV—dipeptidyl peptidase IV; FFAR2/FFAR3—free fatty acid receptors 2 and 3; GaELNs, garlic exosome-like nanoparticles; GIP—glucose-dependent insulinotropic polypeptide; GLP-1—glucagon-like peptide-1; GPCR—G protein-coupled receptor; GPRC6A—G protein-coupled receptor family C group 6 member A; HFD—high-fat diet; IRS2, insulin receptor substrate-2; ITFs—inulin-type fructans; L-cells, enteroendocrine L-cells; PYY—peptide YY; RCTs, randomized controlled trials; RS—resistant starch; SCFAs—short-chain fatty acids; STZ—streptozotocin; T2DM—type 2 diabetes mellitus; TRPA1—transient receptor potential ankyrin 1; — not reported or not applicable.

**Table 3:** Fermented foods and postbiotics as nutritional modulators of endogenous GLP-1 secretion: mechanisms of action and metabolic outcomes.

Fermented Food/Postbiotic System	Model System	Mechanisms Relevant to Endogenous GLP-1 Secretion	Preclinical Outcomes	Clinical Outcomes	References
Kefir and fermented milk products	db/db mouse models; in vitro macrophage assays; RCTs in type 2 diabetes	SCFA, EPS and bioactive peptide formation; improved intestinal barrier; suppression of TLR4/NF-κB signalling; enrichment of butyrate-producing taxa; stimulation of L-cells in murine and in vitro models	Improved glucose tolerance in *db/db* mice; reduced inflammatory signalling; enhanced microbial ecology supporting incretin pathways	Reduced HOMA-IR; improved glycemic control; decreased systemic inflammation in RCTs	[196,197,198,199,200]
Kimchi and LAB-fermented vegetables	High-fat diet mouse models; adipocyte cell culture; human interventions in obesity and pre-diabetes	Organic acids, peptides and γ-PGA; microbiota modulation; increased SCFA formation; regulation of gut–brain signalling; activation of nutrient-sensing pathways in murine and cellular models	Reduced body weight and inflammatory cytokines; improved glucose tolerance in HFD mice	Improved glycemic parameters; favorable microbiota shifts in human studies	[201,202,203,204,205]
Fermented plant-derived foods (tempeh, miso, natto, kombucha, sourdough)	Animal studies; human interventions; narrative reviews	Microbial β-glucosidases, esterases and tannases generating bioactive aglycones; organic acids slowing gastric emptying; postbiotic peptide formation; modulation of nutrient-sensing pathways in animal and in vitro systems	Improved glucose tolerance and insulin sensitivity in animal models; enhanced early GLP-1 responses	Attenuated postprandial glycemia; improved insulin sensitivity, especially for natto (γ-PGA) and sourdough in human studies	[189,206,207]
Probiotic fermented camel milk	Diabetic rodent models; RCT in adolescents with metabolic syndrome	Increased SCFA formation; modulation of gut hormone secretion; improved insulin signalling; enhanced L-cell activity in rodent models	Reduced fasting glucose; improved glucose tolerance; decreased inflammatory biomarkers in rodents	Reduced insulin resistance; improved metabolic profile in adolescents	[197,208]
Lactobacillus-fermented yoghurt	Diabetic animal models; human appetite studies; in vitro fermentation	Bioactive peptide generation; increased viscosity; delayed gastric emptying; stimulation of GLP-1 and PYY in animal and in vitro models	Improved glucose tolerance; reduced lipid accumulation in rodents	Enhanced satiety signalling; favorable postprandial hormone responses in human studies	[209,210,211]
Composite probiotic formulations	db/db mouse models; macrophage polarization assays	Microbiota restructuring; increased SCFA formation; modulation of macrophage M1/M2 balance; enhanced GLP-1 secretion in murine and cellular models	Improved glucose tolerance; reduced inflammatory responses; enhanced insulin secretion in db/db mice	No clinical trials available	[198,212]
Fermented cereal products (barley kernel, sourdough bread)	RCTs in healthy adults; postprandial hormone profiling	Increased SCFA formation; delayed starch digestion; increased luminal viscosity; enhanced GLP-1/PYY secretion in human studies	No animal models reported	Reduced postprandial glucose and insulin; improved appetite regulation in RCTs	[127,206]

Abbreviations: SCFA—short-chain fatty acid; EPS—exopolysaccharide; LAB—lactic acid bacteria; γ-PGA—γ-polyglutamic acid; GLP-1—glucagon-like peptide-1; PYY—peptide YY; L-cells—enteroendocrine L-cells; T2D—type 2 diabetes mellitus; HFD—high-fat diet; HOMA-IR—homeostatic model assessment of insulin resistance; RCTs—randomized controlled trials; TLR4—Toll-like receptor 4; NF-κB—nuclear factor kappa B.

**Table 4:** Polyphenols and plant bioactives modulating endogenous GLP-1 secretion and incretin-related metabolic pathways.

Polyphenol Source/Plant Bioactive	Model System	Mechanisms Relevant to GLP-1 Signalling	Preclinical Outcomes	Clinical Outcomes	References
General dietary polyphenols (fruits, vegetables, mixed plant sources)	In vitro assays; animal models; human studies (reviewed)	Stimulation of L-cell GLP-1 secretion; DPP-4 inhibition; enhancement of insulin signalling	Improved glycemic control and metabolic parameters in animal and cellular models	Improved glycemic regulation and reduced metabolic risk in human studies	[244]
Poorly absorbed intestinal polyphenols (tea, cocoa, berries)	Reviews of intestinal metabolism; evidence from cellular and animal studies	Delivery to distal intestine; direct L-cell stimulation; microbiota-dependent biotransformation	Enhanced GLP-1 secretion; reduced hyperglycemia; improved satiety in preclinical models	Improved vascular function and incretin response in human studies	[270]
Polyphenols and gut microbiota	Review summarizing cellular and animal evidence	Regulation of GLP-1 secretion; DPP-4 inhibition; modulation of microbial metabolites	Improved glucose homeostasis and obesity-related outcomes in animal models	Evidence suggests improved metabolic regulation in human observational studies	[245]
Foxtail millet polyphenols (ferulic-acid-rich fraction)	In vitro L-cell assays; diet-induced obese mouse model	Direct stimulation of endogenous GLP-1 secretion	Improved glucose and lipid metabolism; reduced body-weight gain in mice	- (no clinical trials available)	[271]
Coffee polyphenols and chlorogenic acid	Enteroendocrine cell models; mouse studies; Parkinson’s disease model	cAMP-dependent GLP-1 stimulation independent of GPR119; chlorogenic acid as GLP-1 secretagogue	Improved glycemic control; enhanced insulin sensitivity; neuroprotection in animal models	- (limited clinical evidence; human data limited to signalling studies)	[249,267]
Cacao polyphenols	Mouse studies evaluating circadian metabolism	Regulation of circadian clock genes via GLP-1-dependent signalling	Improved circadian metabolic regulation and glucose homeostasis in mice	- (no clinical trials available)	[266]
Yerba maté	Human studies; investigations describing signalling pathways	Microbial metabolism of ferulic acid enhances GLP-1 signalling	Enhanced incretin response in cellular and animal models	Improved metabolic regulation in human studies	[258]
Black soybean seed coat polyphenols	Rat model assessing GLP-1, cAMP, endothelial signalling	Increased GLP-1 secretion; cAMP-dependent eNOS phosphorylation and nitric oxide synthesis	Improved endothelial function and vascular protection in rats	- (no human studies available)	[264]
Green tea polyphenols and fermented tea metabolites	Enteroendocrine cell models; clinical trial in T2DM; fermented tea studies	Direct L-cell stimulation; improved intestinal barrier; teadenol A activates GPR120 to promote GLP-1 secretion	Enhanced GLP-1 secretion; improved insulin sensitivity in preclinical models	Improved satiety, GLP-1 secretion and barrier function in T2DM clinical trial	[247,259,272,273]
Turmeric and curcumin	L-cell models; animal studies; investigations describing signalling pathways; human interventions	Activation of Ca^2+^/calmodulin-dependent kinase II; microbiota–bile acid–TGR5/FXR signalling; DPP-4 inhibition	Enhanced GLP-1 secretion; improved glucose tolerance; increased satiety in animal and cellular models	Improved glycemic control in human interventions	[159,250,251,262,268,269,274]
Berry and grape polyphenols (ellagitannins, resveratrol, grape seed procyanidins)	In vitro, in vivo and signalling-focused studies	DPP-4 inhibition; modulation of bitter taste receptors; enhancement of GLP-1 secretion	Improved insulin secretion; glycemic control; anti-inflammatory activity in animal and cellular models	Clinical evidence limited or absent	[252,257,275]
Milk protein–polyphenol conjugates (EGCG–apolactoferrin)	Human NCI-H716 enteroendocrine cell model	Modulation of GLP-1 secretion and satiety signalling	Potential enhancement of GLP-1 activity in cell models	No human studies available	[276]
Plant-derived polyphenols with DPP-4 inhibitory activity (myricetin, medicinal plants)	Enzyme inhibition assays; signalling-focused studies; animal models	DPP-4 inhibition prolonging GLP-1 half-life; modulation of DPP-4/Sirt1 pathway	Improved glycemic control; preserved β-cell function; enhanced incretin activity in animal models	Clinical evidence limited	[261,263,277]

Abbreviations: GLP-1—glucagon-like peptide-1; DPP-4—dipeptidyl peptidase-4; L-cells—enteroendocrine L-cells; cAMP—cyclic adenosine monophosphate; eNOS—endothelial nitric oxide synthase; NO—nitric oxide; GPR119—G protein-coupled receptor 119; GPR120—G protein-coupled receptor 120; FXR—farnesoid X receptor; TGR5—Takeda G protein-coupled receptor 5; EGCG—epigallocatechin gallate; Apo-LF—apolactoferrin; T2DM—type 2 diabetes mellitus.

**Table 5:** Mechanisms by which polyphenols and plant-derived bioactives modulate endogenous GLP-1 secretion and incretin-related metabolic responses.

Dominant Mechanism	Representative Polyphenols/Food Sources	Model System	Mechanisms Relevant to GLP-1 Signalling	Preclinical Outcomes	Clinical Outcomes	References
Direct stimulation of enteroendocrine L-cells	Green tea, turmeric, foxtail millet polyphenols	NCI-H716, GLUTag, STC-1 cell models; animal studies	Direct activation of L-cells via calcium- and cAMP-dependent signalling	Improved glucose tolerance; enhanced insulin secretion; increased satiety in animal and cellular models	No clinical trials directly assessing L-cell stimulation	[247,250,251,271]
Microbiota-dependent biotransformation	Poorly absorbed polyphenols, berries, yerba maté, fermented tea	Human studies; animal models; cellular investigations	Microbial conversion of polyphenols into bioactive metabolites (aglycones, phenolic acids, urolithins, teadenol A) that stimulate GLP-1 secretion	Improved glycemic control; enhanced satiety; reduced obesity risk in animal and cellular models	Improved metabolic regulation in human studies	[258,259,270]
Inhibition of DPP-4 activity	Curcumin, myricetin, grape seed procyanidins, berry polyphenols	Enzyme inhibition assays; animal models; signalling-focused studies	DPP-4 inhibition prolongs endogenous GLP-1 half-life and strengthens incretin signalling	Improved glycemic control; enhanced insulin secretion; preserved β-cell function in animal models	Clinical evidence limited	[257,261,262,263]
cAMP-mediated GLP-1 secretion	Coffee polyphenols, chlorogenic acid	Enteroendocrine cell models; animal studies	Activation of cAMP-dependent pathways independent of GPR119, increasing active GLP-1 secretion	Reduced postprandial hyperglycemia; improved insulin sensitivity; neuroprotective effects in animal models	No dedicated clinical trials	[215,233]
Receptor-mediated signalling	Fermented tea (teadenol A), curcumin	Cell culture models; animal studies	Activation of GPR120 and modulation of TGR5/FXR pathways promoting GLP-1 secretion	Enhanced incretin signalling; improved glucose homeostasis in animal models	Clinical evidence limited	[249,268]
Modulation of vascular GLP-1 signalling	Black soybean seed coat polyphenols	Rat studies	Increased GLP-1 secretion activating cAMP–eNOS signalling and nitric oxide synthesis	Improved endothelial function and vascular protection in rats	No human studies available	[264]
Circadian regulation through GLP-1	Cacao polyphenols	Mouse studies	Regulation of circadian clock gene expression via GLP-1-dependent pathways	Improved circadian regulation of glucose and energy metabolism in mice	No clinical trials available	[266]
Integrated modulation of the incretin axis	Mixed dietary polyphenols (fruits, vegetables, tea, cocoa, grapes, berries)	Reviews summarizing in vitro, animal and clinical evidence	Combined stimulation of GLP-1 secretion, DPP-4 inhibition, improved insulin signalling and modulation of gut microbiota	Improved glycemic control; enhanced insulin sensitivity; reduced obesity in animal and cellular models	Reduced metabolic risk and improved glycemic regulation in human studies	[244,245]

Abbreviations: GLP-1—glucagon-like peptide-1; DPP-4—dipeptidyl peptidase-4; L-cells—enteroendocrine L-cells; GPR119—G protein-coupled receptor 119; GPR120—G protein-coupled receptor 120; cAMP—cyclic adenosine monophosphate; eNOS—endothelial nitric oxide synthase; FXR—farnesoid X receptor; TGR5—Takeda G protein-coupled receptor 5.

**Table 6:** Omega-3 fatty acids as nutritional modulators of endogenous GLP-1 secretion through GPR120 (FFAR4)-dependent signalling.

Omega-3 Source/Intervention	Model System	Mechanisms Relevant to GLP-1 and GPR120	Preclinical Outcomes	Clinical Outcomes	References
Marine omega-3 fatty acids (EPA, DHA)	Cellular signalling studies; receptor biology; animal models; human studies	EPA/DHA act as endogenous ligands of GPR120; β-arrestin-2 recruitment suppresses TAK1, NF-κB and JNK pathways, reducing inflammatory cytokine production	Improved insulin sensitivity; reduced inflammation; enhanced metabolic conditions supporting GLP-1 action in animal and cellular models	Improved metabolic profile and reduced inflammatory tone in human studies	[100,101,282]
Fish oil supplementation	Dietary interventions in rodents	Increased intestinal FFAR4 expression; enhanced GLP-1 secretion via calcium-dependent signalling in L-cells; reduced TNF-α expression	Improved intestinal function; increased endogenous GLP-1 release; improved glucose homeostasis in rodents	No clinical trials available	[250]
Omega-3-mediated intestinal barrier protection	Cell culture models; animal studies	GPR120 activation suppresses epithelial inflammatory pathways and strengthens intestinal barrier integrity	Reduced intestinal inflammation; improved nutrient sensing and enteroendocrine signalling in preclinical models	No clinical trials available	[284,286]
Marine omega-3 supplementation in obesity (EPICO trial)	Randomized controlled clinical trial	Increased FFAR4 activation in peripheral blood mononuclear cells; attenuation of systemic inflammatory responses	Preclinical data not included in EPICO	Reduced inflammatory biomarkers; improved metabolic profile in obesity	[287]
GPR120 signalling in systemic inflammation	Reviews summarizing cellular, animal and human evidence	FFAR4 activation regulates inflammatory pathways in adipose tissue, immune cells and intestinal epithelium	Reduced inflammation; improved insulin responsiveness in animal and cellular models	Improved inflammatory profile in human studies	[288,289]
Biased agonism of GPR120	Structural studies; pharmacological investigations	Natural omega-3 ligands activate balanced β-arrestin- and G-protein-dependent signalling, avoiding excessive receptor stimulation	Physiological incretin stimulation with reduced risk of receptor desensitization in preclinical models	No clinical trials available	[290]
Omega-3 fatty acids as nutritional GLP-1 modulators	Evidence integrating cellular, animal and clinical studies	Activation of GPR120 in L-cells, adipocytes, macrophages and intestinal epithelium; enhanced endogenous GLP-1 secretion together with anti-inflammatory signalling	Improved glucose tolerance; enhanced insulin sensitivity; improved appetite regulation in animal and cellular models	Improved cardiometabolic profile in human studies	[101,287,289]

Abbreviations: EPA—eicosapentaenoic acid; DHA—docosahexaenoic acid; FFAR4—free fatty acid receptor 4 (GPR120); GLP-1—glucagon-like peptide-1; GPCR—G-protein-coupled receptor; JNK—c-Jun *N*-terminal kinase; NF-κB—nuclear factor kappa B; PBMCs—peripheral blood mononuclear cells; TAK1—transforming growth factor-β-activated kinase 1; TNF-α—tumour necrosis factor-α.

**Table 7:** Biological determinants of interindividual variability in endogenous GLP-1 responsiveness and implications for precision nutrition.

Determinant	Primary Mechanism Influencing GLP-1 Secretion	Consequences for Metabolic Response	Implications for Precision Nutrition	References
Gut microbiota composition (enterotypes)	Differences in SCFA production, bile acid metabolism, microbial fermentation and postbiotic formation regulate FFAR2/3-, TGR5- and GPR119-mediated GLP-1 secretion	Variable satiety, glycemic control, insulin sensitivity and body-weight response	Microbiome profiling may identify individuals most likely to benefit from fermentable fibers, polyphenols and fermented foods	[46,47,48,77,80]
Enteroendocrine L-cell function	Individual variation in L-cell density, receptor abundance (FFAR2/3, FFAR4, GPR119, TGR5), intracellular Ca^2+^ signalling and secretory capacity	Different magnitudes of endogenous GLP-1 release after identical dietary stimuli	Identification of responder phenotypes may improve personalized dietary recommendations	[22,52,53]
Genetic variation	Polymorphisms affecting nutrient-sensing receptors (FFAR4, FFAR2/3, GPR119, TGR5) alter ligand binding and downstream signalling	Heterogeneous responses to dietary lipids, SCFAs and protein-derived metabolites	Genotype-informed nutritional interventions may optimize endogenous incretin responses	[103,108,115,116]
Bile acid metabolism	Individual differences in bile acid pool composition regulate TGR5 activation and enteroendocrine GLP-1 secretion	Differences in glucose homeostasis, lipid metabolism and postprandial hormone responses	Dietary strategies modifying bile acid metabolism may enhance incretin signalling	[86,91,92,97]
Intestinal epithelial integrity	Barrier function and epithelial inflammation regulate nutrient access to enteroendocrine L-cells and receptor activation	Altered GLP-1 secretion and reduced metabolic responsiveness during intestinal dysfunction	Interventions targeting epithelial health (dietary fibre, fermented foods, postbiotics) may improve incretin responsiveness	[19,45,167]
Neural regulation	Vagal afferents and sympathetic–enteroendocrine signalling modulate nutrient sensing and GLP-1 secretion	Individual variability in appetite regulation, gastric emptying and glycemic control	Neural regulation should be considered in personalised metabolic interventions	[5,52]
Host metabolic phenotype	Obesity, insulin resistance, mitochondrial dysfunction and chronic low-grade inflammation alter enteroendocrine responsiveness	Reduced GLP-1 secretion and diminished metabolic flexibility	Baseline metabolic health influences effectiveness of nutritional GLP-1-enhancing strategies	[52,55]
Dietary patterns	Habitual intake of dietary fiber, polyphenols, omega-3 fatty acids, fermented foods and protein-derived peptides shapes microbiota composition and receptor activation	Long-term adaptation of endogenous incretin secretion and metabolic homeostasis	Personalized dietary patterns may maximize endogenous GLP-1 activity	[101,143,177,183]
Multi-omics integration	Combined analysis of microbiome, metabolome, host genetics and dietary intake improves prediction of GLP-1 responsiveness	Improved prediction of responder and non-responder phenotypes	Foundation for precision nutrition and personalized dietary modulation of endogenous GLP-1	[51,80,293]

Abbreviations: FFAR2/3—free fatty acid receptors 2 and 3; FFAR4—free fatty acid receptor 4 (GPR120); GPR119—G-protein-coupled receptor 119; GLP-1—glucagon-like peptide-1; L-cells—enteroendocrine L-cells; SCFAs—short-chain fatty acids; TGR5—Takeda G-protein-coupled receptor 5.

**Table 8:** Perspectives of dietary GLP-1 modulation in precision nutrition.

Clinical Area	Dietary Strategy	Main GLP-1-Related Mechanism	Potential Clinical Application	Level of Evidence
Obesity	Fermentable fibres	SCFA → FFAR2/3 → GLP-1	Weight management	Human RCTs + meta-analyses (high-quality clinical evidence demonstrating effects on satiety, glycemia and body weight)
Type 2 diabetes	Polyphenols	GLP-1 secretion + DPP-4 inhibition	Glycemic control	Human + animal (supported by clinical studies and in vivo models demonstrating effects on GLP-1 signalling and glycemic outcomes)
Metabolic syndrome	Fermented foods/postbiotics	Microbiota remodelling	Improved insulin sensitivity	Human + animal (supported by clinical studies and in vivo models showing microbiota-dependent improvements in insulin sensitivity)
Insulin resistance	Protein hydrolysates	GPR119-mediated incretin secretion	Improved postprandial glycemia	Mainly preclinical (supported by animal and in vitro studies demonstrating GPR119-dependent incretin effects, with no direct human data)
Chronic inflammation	EPA/DHA	GPR120 activation	Reduced inflammation, improved GLP-1 signalling	Human + animal (supported by clinical studies and in vivo models showing anti-inflammatory effects and enhanced GLP-1 signalling)
Precision nutrition	Multi-omics-guided diet	Personalized GLP-1 responsiveness	Individualized nutritional therapy	Emerging evidence (supported by early multi-omics studies indicating potential for personalized incretin profiling, but requiring further validation)

Abbreviations: GLP-1—glucagon-like peptide-1; SCFAs—short-chain fatty acids; FFAR2/3—free fatty acid receptors 2 and 3; GPR119—G protein-coupled receptor 119; GPR120 (FFAR4)—G protein-coupled receptor 120 (free fatty acid receptor 4); DPP-4—dipeptidyl peptidase-4; EPA—eicosapentaenoic acid; DHA—docosahexaenoic acid; RCTs—randomized controlled trials.

## Data Availability

No new data were created or analyzed in this study. Data sharing is not applicable to this article. The data presented in this study are available within the article.

## Abbreviations

The following abbreviations are used in this manuscript:

SCFA	short chain fatty acids
RS	resistant starch
AXOS	arabinoxylan oligosaccharides
DF	dietary fiber
GLP 1	glucagon like peptide 1
PYY	peptide YY
GIP	glucose-dependent insulinotropic polypeptide
ESG	enzymatically synthesized glycogen
NPF	net portal flux
FFA	free fatty acids
POMC	proopiomelanocortin
LN	lean
OWO	overweight/obese
IL 6	interleukin 6
TAG	triacylglycerides
CCK	cholecystokinin
NSP	non starch polysaccharides
BA	bile acids
FFAR2/3	free fatty acid receptor 2/3
GLP 2	glucagon like peptide 2
TG	triglycerides
ITF	inulin type fructans
L cells	enteroendocrine L cells
GE	gastric emptying
iAUC	incremental area under the curve
DPP IV	dipeptidyl peptidase IV
HFD	High-fat diet
T2DM	type 2 diabetes mellitus
TC	total cholesterol
LDL C	low density lipoprotein cholesterol
HDL C	high density lipoprotein cholesterol
STZ	streptozotocin
REPH	rice endosperm protein hydrolysate
RBPH	rice bran protein hydrolysate
LWP	low molecular wheat protein hydrolysate
GPCR	G protein coupled receptor
AMPK	AMP activated protein kinase
IRS2	insulin receptor substrate 2
Akt	protein kinase B
TRPA1	transient receptor potential ankyrin 1
ELNs	exosome like nanoparticles
OMVs	outer membrane vesicles
HTL	Helianthus tuberosus
CKJ	chungkookjang
CQA	caffeoylquinic acids
EPS	exopolysaccharides
LAB	lactic acid bacteria
γ PGA	gamma polyglutamic acid
T2D	type 2 diabetes
HOMA IR	homeostatic model assessment of insulin resistance
DPP 4	dipeptidyl peptidase 4
CPE	coffee polyphenol extract
FMP	foxtail millet polyphenols
cAMP	cyclic adenosine monophosphate
NO	nitric oxide
eNOS	endothelial nitric oxide synthase
FXR	farnesoid X receptor
TGR5	Takeda G protein coupled receptor 5
EGCG	epigallocatechin gallate
Apo LF	apolactoferrin
